# Tales of diversity: Genomic and morphological characteristics of forty-six *Arthrobacter* phages

**DOI:** 10.1371/journal.pone.0180517

**Published:** 2017-07-17

**Authors:** Karen K. Klyczek, J. Alfred Bonilla, Deborah Jacobs-Sera, Tamarah L. Adair, Patricia Afram, Katherine G. Allen, Megan L. Archambault, Rahat M. Aziz, Filippa G. Bagnasco, Sarah L. Ball, Natalie A. Barrett, Robert C. Benjamin, Christopher J. Blasi, Katherine Borst, Mary A. Braun, Haley Broomell, Conner B. Brown, Zachary S. Brynell, Ashley B. Bue, Sydney O. Burke, William Casazza, Julia A. Cautela, Kevin Chen, Nitish S. Chimalakonda, Dylan Chudoff, Jade A. Connor, Trevor S. Cross, Kyra N. Curtis, Jessica A. Dahlke, Bethany M. Deaton, Sarah J. Degroote, Danielle M. DeNigris, Katherine C. DeRuff, Milan Dolan, David Dunbar, Marisa S. Egan, Daniel R. Evans, Abby K. Fahnestock, Amal Farooq, Garrett Finn, Christopher R. Fratus, Bobby L. Gaffney, Rebecca A. Garlena, Kelly E. Garrigan, Bryan C. Gibbon, Michael A. Goedde, Carlos A. Guerrero Bustamante, Melinda Harrison, Megan C. Hartwell, Emily L. Heckman, Jennifer Huang, Lee E. Hughes, Kathryn M. Hyduchak, Aswathi E. Jacob, Machika Kaku, Allen W. Karstens, Margaret A. Kenna, Susheel Khetarpal, Rodney A. King, Amanda L. Kobokovich, Hannah Kolev, Sai A. Konde, Elizabeth Kriese, Morgan E. Lamey, Carter N. Lantz, Jonathan S. Lapin, Temiloluwa O. Lawson, In Young Lee, Scott M. Lee, Julia Y. Lee-Soety, Emily M. Lehmann, Shawn C. London, A. Javier Lopez, Kelly C. Lynch, Catherine M. Mageeney, Tetyana Martynyuk, Kevin J. Mathew, Travis N. Mavrich, Christopher M. McDaniel, Hannah McDonald, C. Joel McManus, Jessica E. Medrano, Francis E. Mele, Jennifer E. Menninger, Sierra N. Miller, Josephine E. Minick, Courtney T. Nabua, Caroline K. Napoli, Martha Nkangabwa, Elizabeth A. Oates, Cassandra T. Ott, Sarah K. Pellerino, William J. Pinamont, Ross T. Pirnie, Marie C. Pizzorno, Emilee J. Plautz, Welkin H. Pope, Katelyn M. Pruett, Gabbi Rickstrew, Patrick A. Rimple, Claire A. Rinehart, Kayla M. Robinson, Victoria A. Rose, Daniel A. Russell, Amelia M. Schick, Julia Schlossman, Victoria M. Schneider, Chloe A. Sells, Jeremy W. Sieker, Morgan P. Silva, Marissa M. Silvi, Stephanie E. Simon, Amanda K. Staples, Isabelle L. Steed, Emily L. Stowe, Noah A. Stueven, Porter T. Swartz, Emma A. Sweet, Abigail T. Sweetman, Corrina Tender, Katrina Terry, Chrystal Thomas, Daniel S. Thomas, Allison R. Thompson, Lorianna Vanderveen, Rohan Varma, Hannah L. Vaught, Quynh D. Vo, Zachary T. Vonberg, Vassie C. Ware, Yasmene M. Warrad, Kaitlyn E. Wathen, Jonathan L. Weinstein, Jacqueline F. Wyper, Jakob R. Yankauskas, Christine Zhang, Graham F. Hatfull

**Affiliations:** 1 Biology Department, University of Wisconsin-River Falls, River Falls, Wisconsin, United States of America; 2 Department of Biological Sciences, University of Pittsburgh, Pittsburgh, Pennsylvania, United States of America; 3 Department of Biology, Baylor University, Waco, Texas, United States of America; 4 Department of Science, Cabrini University, Radnor, Pennsylvania, United States of America; 5 Biology Department, Western Kentucky University, Bowling Green, Kentucky, United States of America; 6 Department of Biological Sciences, University of North Texas, Denton, Texas, United States of America; 7 Center for Life Sciences Education, The Ohio State University, Columbus, Ohio, United States of America; 8 Biology Department, Saint Joseph’s University, Philadelphia, Pennsylvania, United States of America; 9 Biology Department, Bucknell University, Lewisburg, Pennsylvania, United States of America; 10 Department of Biological Sciences, Carnegie Mellon University, Pittsburgh, Pennsylvania, United States of America; 11 Biological Sciences, Lehigh University, Bethlehem, Pennsylvania, United States of America; UNITED STATES

## Abstract

The vast bacteriophage population harbors an immense reservoir of genetic information. Almost 2000 phage genomes have been sequenced from phages infecting hosts in the phylum Actinobacteria, and analysis of these genomes reveals substantial diversity, pervasive mosaicism, and novel mechanisms for phage replication and lysogeny. Here, we describe the isolation and genomic characterization of 46 phages from environmental samples at various geographic locations in the U.S. infecting a single *Arthrobacter* sp. strain. These phages include representatives of all three virion morphologies, and Jasmine is the first sequenced podovirus of an actinobacterial host. The phages also span considerable sequence diversity, and can be grouped into 10 clusters according to their nucleotide diversity, and two singletons each with no close relatives. However, the clusters/singletons appear to be genomically well separated from each other, and relatively few genes are shared between clusters. Genome size varies from among the smallest of siphoviral phages (15,319 bp) to over 70 kbp, and G+C contents range from 45–68%, compared to 63.4% for the host genome. Although temperate phages are common among other actinobacterial hosts, these *Arthrobacter* phages are primarily lytic, and only the singleton Galaxy is likely temperate.

## Introduction

The bacteriophage population is vast, dynamic, old, and highly diverse genetically [1]. The majority of the reference-sequenced bacteriophages in the GenBank database [2] correspond to just five host phyla, the Actinobacteria, Bacteroidetes, Cyanobacteria, Firmicutes, and Proteobacteria. Within the Actinobacteria, most of the phages were isolated on *Mycobacterium smegmatis* mc^2^155 (http://www.phagesdb.org), with smaller numbers on *Gordonia* [3, 4], *Nocardia* [5], *Rhodococcus* [6, 7] *Streptomyces* [8], and *Tsukamurella* [9] hosts. Comparative genomic analyses of 627 mycobacteriophages showed them to span considerable genetic variability reflecting a continuum of diversity but with highly uneven representation of different genomic types [10]. This contrasts with comparative genomics of 142 cyanobacteriophages grouped into ten lineages, which appear as discrete genetic populations [11].

To further investigate the genetic diversity of phages infecting Actinobacterial hosts, we explored the use of *Arthrobacter* sp. for the isolation of phages from environmental samples. *Arthrobacter* spp. are primarily soil organisms, some of which break down complex hydrocarbons, including hexavalent chromium, 4-chlorophenol, and various aromatic compounds such as pyridine and its derivatives; as such, they may have potential for use in bioremediation [12–14]. *Arthrobacter* spp. including *A*. *arilaitensis* are also components of smear-ripened cheese [15], and some *Arthrobacter* strains produce antibacterials such as penicillin derivatives [16]. *Arthrobacter* cells lack mycolic acids, and stain as gram-variable related to a transition from coccus to rod morphology during cell growth [17].

Several phages of *Arthrobacter* hosts have been isolated and used for bacterial strain typing [18–22] although only two have been sequenced: vB_ArS-ArV2 (ArV2) [23] and vB_ArtM-ArV1(ArV1) [24], both isolated on the environmental strain *Arthrobacter sp*. 68b. Here we describe the isolation and characterization of 46 phages infecting *Arthrobacter sp*. ATCC 21022 [25]. They are genomically diverse, but share no nucleotide sequence similarity with other phages infecting actinobacterial hosts including the mycobacteriophages.

## Results and discussion

### Arthrobacter phage isolation

Forty-six phages were isolated from soil samples using *Arthrobacter sp*. ATCC21022 as host (Table 1), one of which (Gordon) was isolated by direct plating of processed environmental samples onto an *Arthrobacter* lawn. The others were obtained by enrichment as described previously [26]. Phages were isolated by students in the Phage Hunters Integrating Research and Education (PHIRE) [27] at the University of Pittsburgh and Science Education Alliance-Phage Hunters Advancing Genomics and Evolutionary Science (SEA-PHAGES) [28] program, from nine institutions: Baylor University, Bucknell University, Cabrini University, Carnegie Mellon University, Lehigh University, Saint Joseph’s University, University of North Texas, Western Kentucky University, and University of Wisconsin-River Falls. Most phages were isolated from samples collected near these universities (S1 Fig and S1 Table). Phages were identified as plaques on lawns of *Arthrobacter* ATCC 21022, plaque purified, amplified, and genomic DNA was extracted as described previously [29]. All of the phages form clear plaques, with the exception of Galaxy that forms turbid plaques.

**Table 1:** Forty-six Arthrobacter phages.

Phage	Cluster	GenBank Acc. no.	Genome Length(bp)	%GC	# genes	Virion Morphology	Structure of Genome Ends	Location
Bennie	AK	KU160640	43,075	61.4	62	Sipho	13 base 3'	South Park, PA
DrRobert	AK	KU160643	42,601	60.6	59	Sipho	13 base 3'	Pittsburgh, PA
Glenn	AK	KU160645	44,389	60.8	64	Sipho	13 base 3'	Pittsburgh, PA
HunterDalle	AK	KU160648	43,336	61.6	60	Sipho	13 base 3'	Laurel Springs, NJ
Immaculata	AK	KU160649	43,661	61	62	Sipho	13 base 3'	Immaculata, PA
Joann	AK	KU160652	44,183	60.7	63	Sipho	13 base 3'	Clayton, OK
Korra	AK	KU160653	43,707	61.1	60	Sipho	13 base 3'	Bethel Park, PA
Preamble	AK	KU160659	43,374	60.7	64	Sipho	13 base 3'	Radnor, PA
Pumancara	AK	KU160661	42,830	61.7	61	Sipho	13 base 3'	Pittsburgh, PA
RAP15	AK	KU160662	44,259	60.9	63	Sipho	13 base 3'	Pittsburgh, PA
Vulture	AK	KU160671	43,336	61.1	64	Sipho	13 base 3'	Marlton, NJ
Wayne	AK	KU160672	44,371	61.1	62	Sipho	13 base 3'	N. Huntingdon, PA
Laroye	AL	KU160654	60,005	64.8	99	Sipho	Circularly permuted	Pittsburgh, PA
Salgado	AL	KU160664	59,807	64.6	99	Sipho	Circularly permuted	Pittsburgh, PA
Circum	AM	KU160642	58,353	45.2	99	Sipho^3^	9 base 3'	Denton, TX
Mudcat	AM	KU647628	59,443	45.1	95	Sipho^3^	9 base 3'	Central City, KY
Decurro	AN	KT355471	15,524	60.2	26	Sipho	11 base 3'	Lewisburg, PA
Jessica	AN	KT355473	15,556	60.1	26	Sipho	11 base 3'	Lewisburg, PA
Maggie	AN	KU160655	15,556	60.1	26	Sipho	11 base 3'	Bethlehem, PA
Moloch	AN	KU160657	15,630	60	26	Sipho	11 base 3'	Pittsburgh, PA
Muttlie	AN	KU160658	15,524	60.2	26	Sipho	11 base 3'	West Chester, PA
Sandman	AN	KT355475	15,630	60	26	Sipho	11 base 3'	Seaside Heights, NJ
Stratus	AN	KU160667	15,630	60	26	Sipho	11 base 3'	Radnor, PA
Toulouse	AN	KU160670	15,319	60.3	25	Sipho	11 base 3'	Hudson, WI
TymAbreu	AN	KT783672	15,556	60.1	26	Sipho	11 base 3'	Hudson, WI
Yank	AN	KU160674	15,524	60.2	26	Sipho	11 base 3'	Lewisburg, PA
Brent	AO	KT365401	49,879	63.4	74	Myo	Circularly permuted	Broomall, PA
Jawnski	AO	KU160651	49,419	63.4	73	Myo	Circularly permuted	Pittsburgh, PA
BarretLemon	AO	KU647629	51,290	60.9	79	Myo	Circularly permuted	Chippewa Falls, WI
Martha	AO	KU160656	51,027	61	77	Myo	Circularly permuted	Pittsburgh, PA
Sonny	AO	KU160665	50,909	61.1	77	Myo	Circularly permuted	Pittsburgh, PA
TaeYoung	AO	KU160668	50,999	61	78	Myo	Circularly permuted	Pittsburgh, PA
Tank	AP	KU160669	67,592	62.9	105	Sipho	589 base Direct terminal rpt^1^	Philadelphia, PA
Wilde	AP	KU160673	68,203	62.9	109	Sipho	589 base Direct terminal rpt	Montclair, NJ
Amigo	AQ	KU160638	59,173	52.9	86	Sipho	1584 Direct terminal rpt	Spring, Texas
Anansi	AQ	KU160639	58,848	53	86	Sipho	1584 Direct terminal rpt	Phoenixville, PA
Gorgeous	AQ	KU160647	58,979	53	86	Sipho	1584 Direct terminal rpt	Lafayette, Hills PA
Rings	AQ	KU160663	59,167	53	86	Sipho	1584 Direct terminal rpt	Radnor, PA
SorJuana	AQ	KU160666	58,979	53	86	Sipho	1584 Direct terminal rpt	Royersford, PA
KellEzio	AT	KU647626	58,871	63.3	99	Sipho	unknown^2^	Burkesville, KY
Kitkat	AT	KU647627	58,560	63.4	100	Sipho	unknown^2^	Greenbrae, CA
CapnMurica	AU	KU160641	58,159	49.6	88	Sipho	9 base 3'	Pittsburgh, PA
Gordon	AU	KU160646	58,279	49.8	89	Sipho	9 base 3'	South Park, PA
PrincessTrina	AR	KU160660	70,265	61.6	112	Myo	Circularly permuted	Laurel Springs, NJ
Galaxy	Singleton	KU160644	37,809	68.4	65	Sipho	12 base 3'	Harmony, PA
Jasmine	Singleton	KU160650	46,723	45.9	58	Podo	1330 Direct terminal rpt	Pittsburgh, PA

^1^right end is clear, left end is ambiguous

^2^95% of sequencing reads align to reverse strand

^3^prolate head

### Virion morphologies

**Fig 1:**
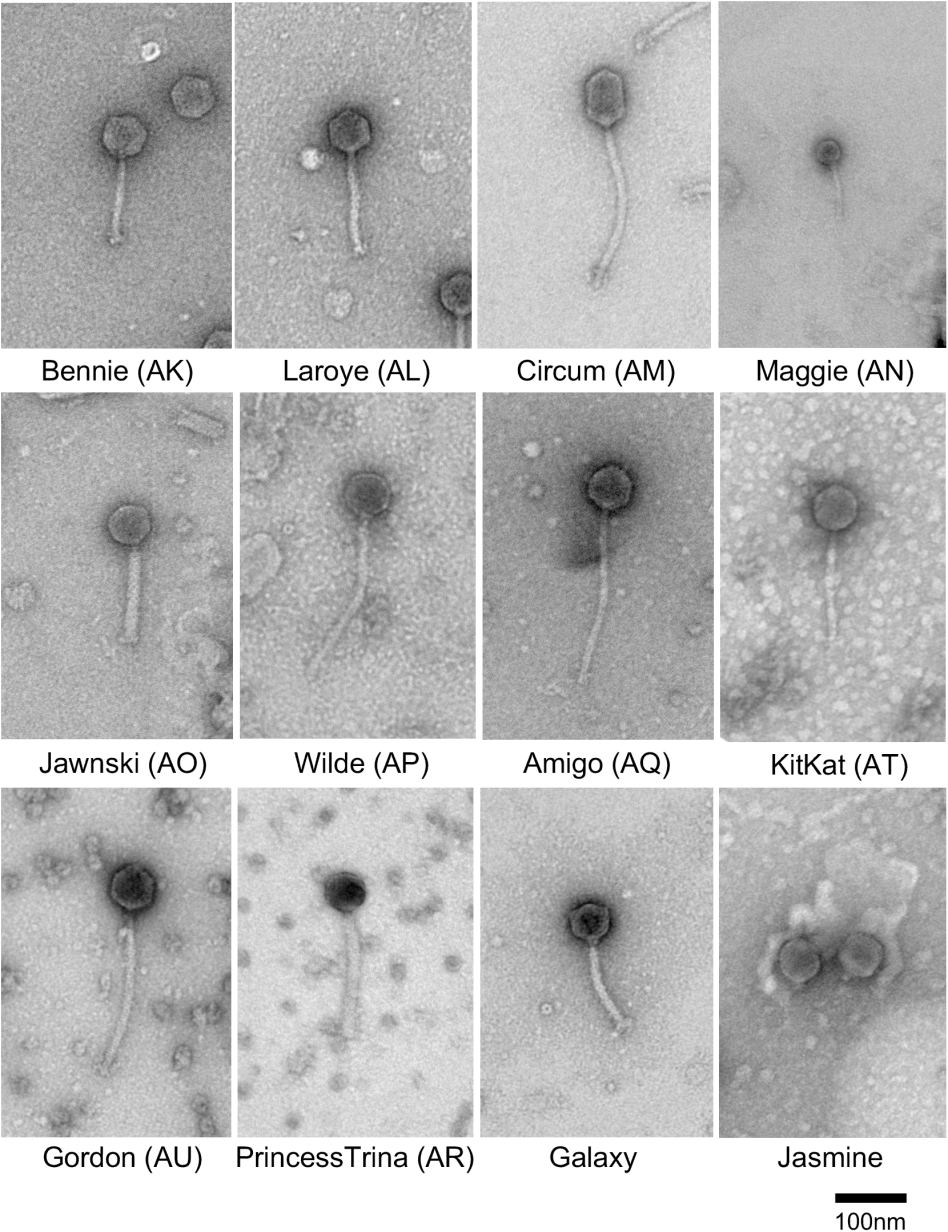
*Arthrobacter* virion morphologies. Electron micrographs of representative *Arthrobacter* phages. Scale bar corresponds to 100 nm.

Phage particles were observed by transmission electron microscopy with negative staining (Fig 1). Most have siphoviral morphologies with non-contractile, flexible tails, ranging in length from 111.2 (± 11.0) to 242.3 (± 13.3) nm, and isometric heads ranging in size from 55.8 (± 4.0) to 61.4 (± 2.4) nm. Two of the siphoviruses (Circum and Mudcat) have prolate heads with length of 73.7 (± 1.3) nm x width of 50.5 (± 2.2) nm (Fig 1, S2 Table). Seven of the phages (Brent, Jawnski, Martha, Sonny, TaeYoung, BarretLemon, and PrincessTrina) have myoviral morphologies with a rigid tail and a tail sheath similar in appearance to P2-like [30] or Mu-like [31] myoviral phages infecting *E*. *coli* and other Enterobacteria. Myoviral phages of other Actinobacterial hosts are less common than siphoviruses but include the Cluster C mycobacteriophages [32] and the singleton *Rhodococcus* phage E3 [33]. Interestingly, Jasmine has a podoviral morphology with a head diameter of 59.8 (± 2.9) nm and a short stubby tail of 10.3 (± 0.9) nm (Fig 1). Two phages of *Arthrobacter* have been previously described with similar morphologies [20] but their genomes have yet to be sequenced, and to our knowledge, these are the only podoviruses of Actinobacterial hosts among over 1,000 sequenced phages that been examined morphologically.

### *Arthrobacter* phage genometrics

The *Arthrobacter* phage genomes were sequenced and putative gene locations and functions were assigned based on bioinformatic analyses as described previously [10, 32, 34]. Genome lengths range considerably, from 15,319 bp (Toulouse) to 70,265 bp (PrincessTrina), with an average genome length of 45,832 bp (Table 1). The G+C contents span a broad range, from 45.1% (Mudcat) to 68.4% (Galaxy), such that the G+C content for many of the phages is substantially different from the *Arthrobacter sp*. ATCC 21022 host (63.4%) [25]. The genome termini vary considerably: many have cohesive ends with 3’ single stranded DNA extensions of 9–13 bases, some are circularly permuted and terminally redundant, and others have a direct terminal repeat ranging from 589 bp to 1584 bp long (Table 1). For two genomes, KellEzio and Kitkat, the ends could not be readily determined, but they are likely circularly permuted (manuscript in preparation). For these and the other circularly permuted terminally redundant genomes the sequences were linearized at positions near the 5’ ends of the predicted terminase genes.

### *Arthrobacter* phage cluster assignments

**Fig 2:**
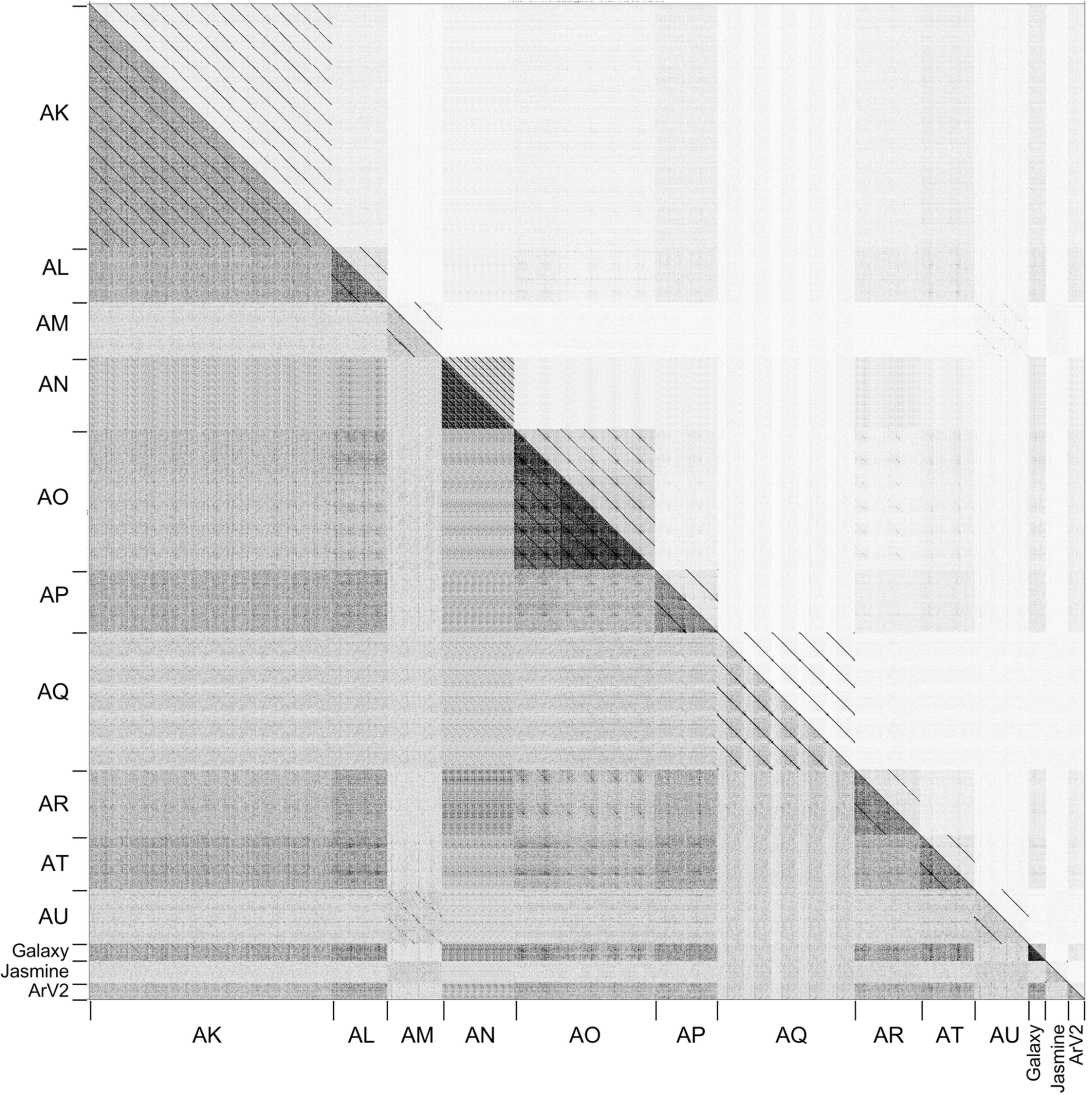
Nucleotide sequence comparison of *Arthrobacter* phages. Dot Plot of *Arthrobacter* phage genomes displayed using Gepard [35]. Individual genome sequences were concatenated into a single file arranged such that related genomes were adjacent to each other. The assignment of clusters is shown along both the left and bottom.

**Fig 3:**
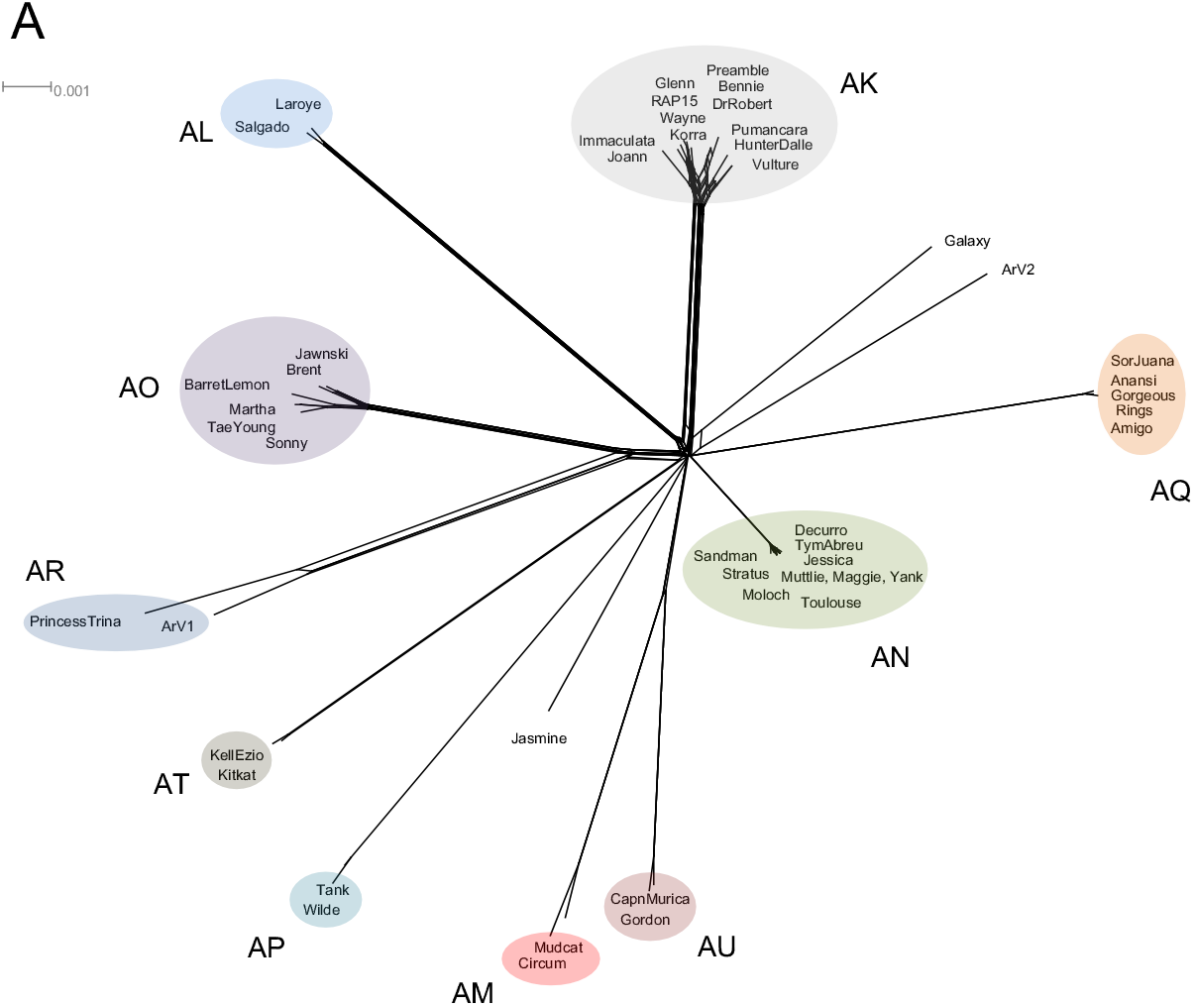
Splitstree representation of *Arthrobacter* phages and average nucleotide comparisons of Cluster AO *Arthrobacter* phages. All *Arthrobacter* phage predicted proteins were assorted into 1052 phams according to shared amino acid sequence similarities. Each genome was then assigned a value reflecting the presence or absence of a pham member, and the genomes were compared and displayed using Splitstree [36]. Cluster and subcluster assignments derived from the dot plot and ANI analyses are annotated. The scale bar indicates 0.001 substitutions/site.

**Fig 4:**
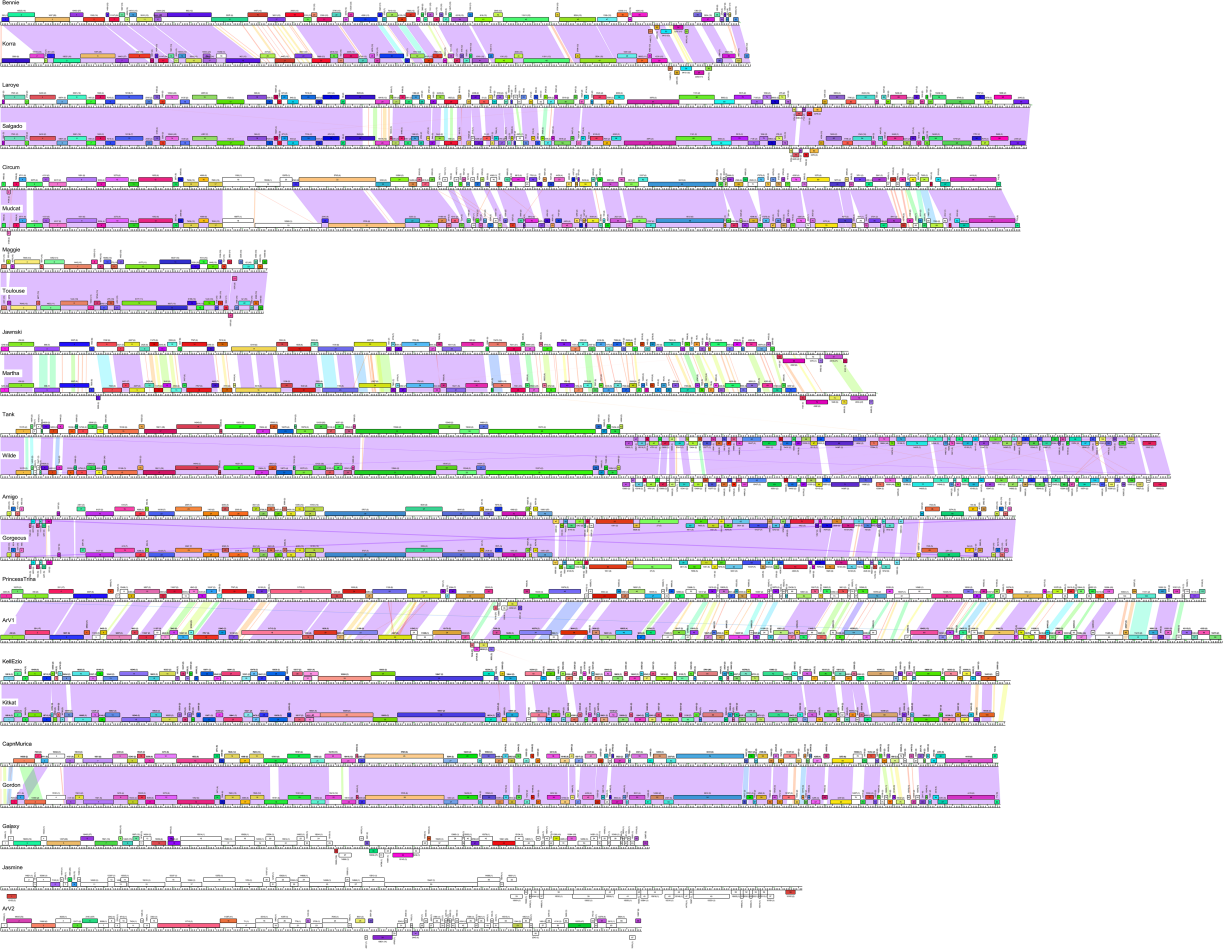
Pairwise alignment of clustered *Arthrobacter* phages. The genomes of 23 *Arthrobacter* phages are shown. Pairwise nucleotide sequence similarity is displayed by color-spectrum coloring between the genomes, with violet as most similar and red as least similar. Genes are shown as boxes above (transcribed rightwards) and below (transcribed leftwards) each genome line; boxes are colored according to the gene phamilies they are assigned [29]. Maps were generated using Phamerator and its database Actinobacteriophage_692.

Dotplot comparison of *Arthrobacter* phage genomes shows distinct lineages with some phages more closely related to some than to others (Fig 2). Using this information, together with a gene content-based phylogeny (Fig 3), average nucleotide identity (ANI) values (S3 Table), pairwise genome alignments (Fig 4) and similar clustering parameters to those described previously [29, 32], these phages group into ten distinct clusters (AK–AU) and two singletons (Galaxy and Jasmine) (Table 1). The previously described phage, ArV1, clusters with PrincessTrina (Cluster AR); phage ArV2 is a singleton (Table 1, Fig 2). We note that phages in Cluster AM and AU share some observable nucleotide similarity in the Dotplot comparison (Fig 2), although their shared ANI values are below 0.6 (S3 Table); they also have a common branch in the network gene-content phylogeny (Fig 2) corresponding to them sharing approximately 30% of their genes using amino acid sequence comparisons. However, they are sufficiently different to warrant grouping into the separate Clusters AM and AU. None of the clusters warrant subdivision based on ANI values (S3 Table).

### *Arthrobacter* phage genome organizations

#### General genomic features

**Fig 5:**
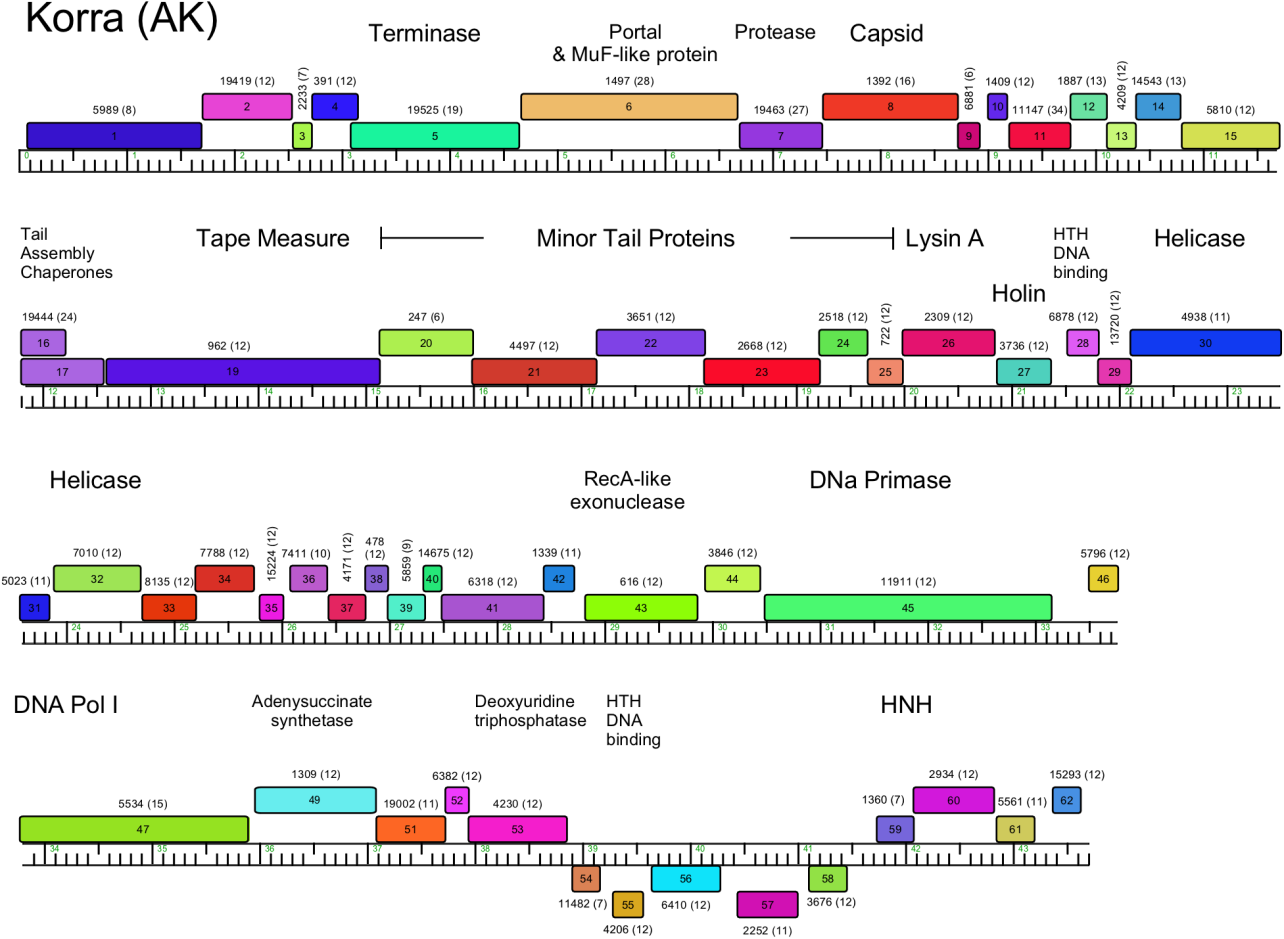
Genome organization of *Arthrobacter* phage Korra, Cluster AK. The genome of *Arthrobacter* phage Korra is shown with predicted genes depicted as boxes either above (rightwards-expressed) or below (leftwards-expressed) the genome. Genes are colored according to the phamily designations using Phamerator and database Actinobacteriophage_692, with the phamily number shown above each gene with the number of phamily members in parentheses.

**Fig 6:**
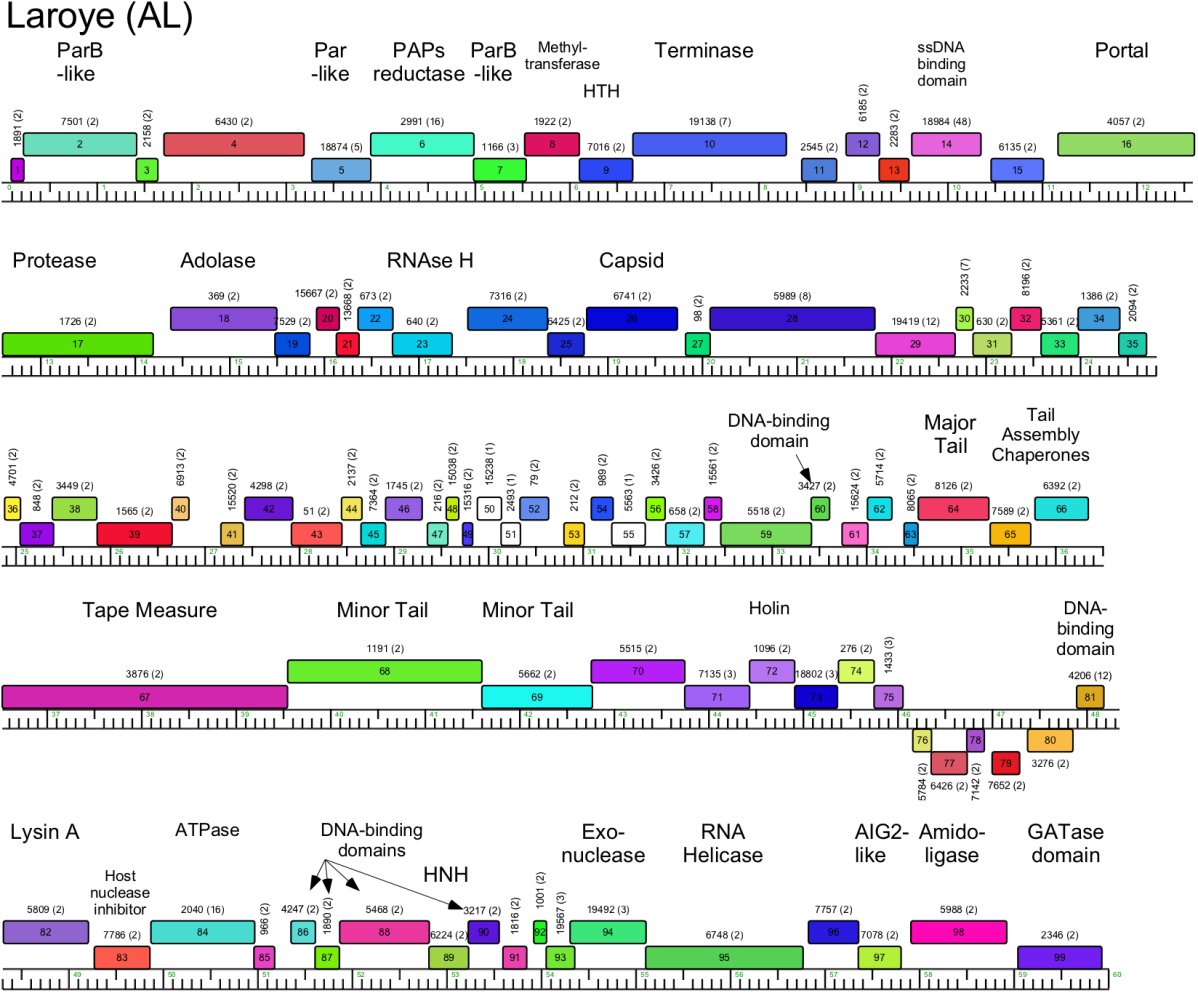
Genome organization of *Arthrobacter* phage Laroye, Cluster AL. See Fig 5 for details.

**Fig 7:**
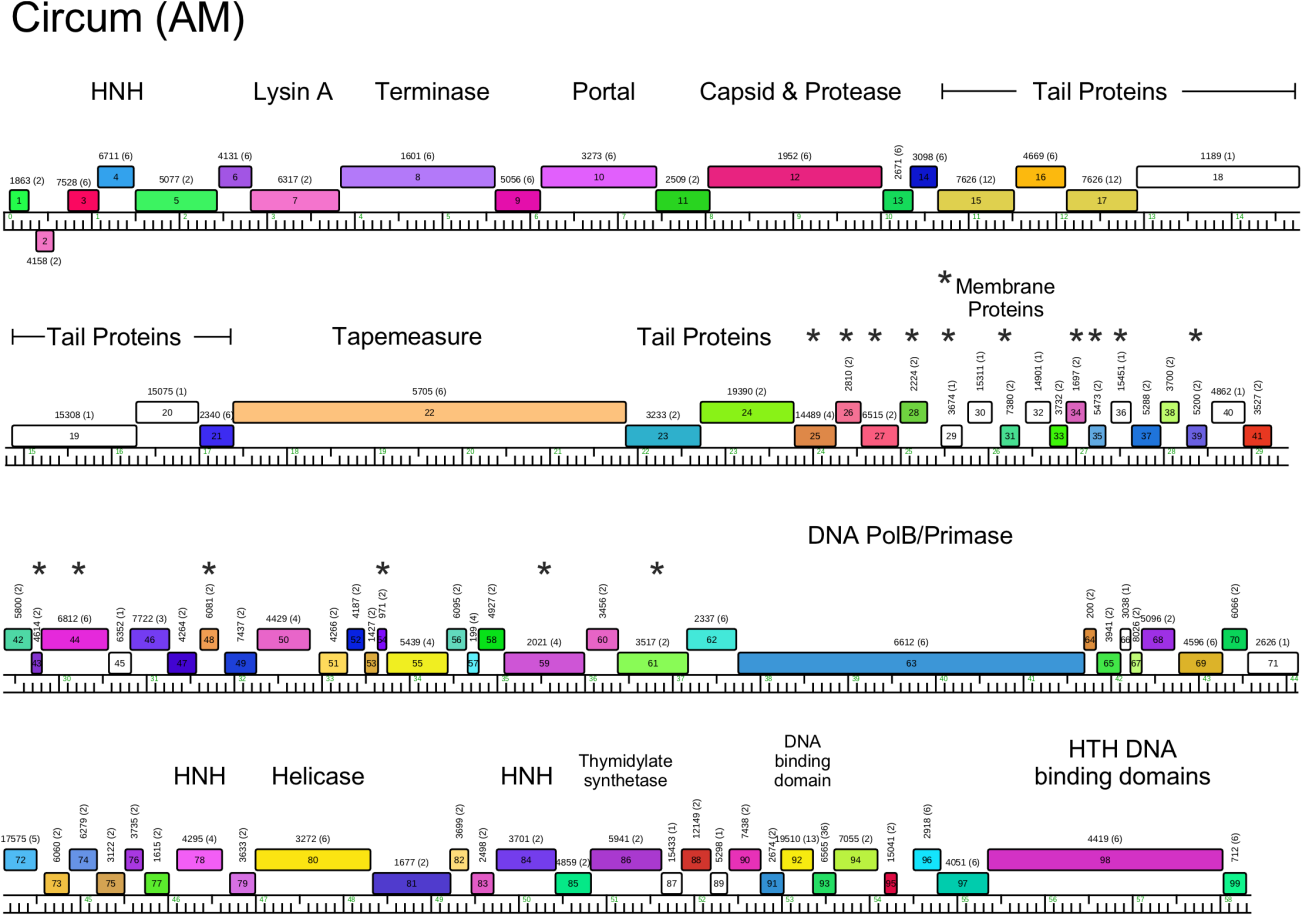
Genome organization of *Arthrobacter* phage Circum, Cluster AM. See Fig 5 for details.

**Fig 8:**
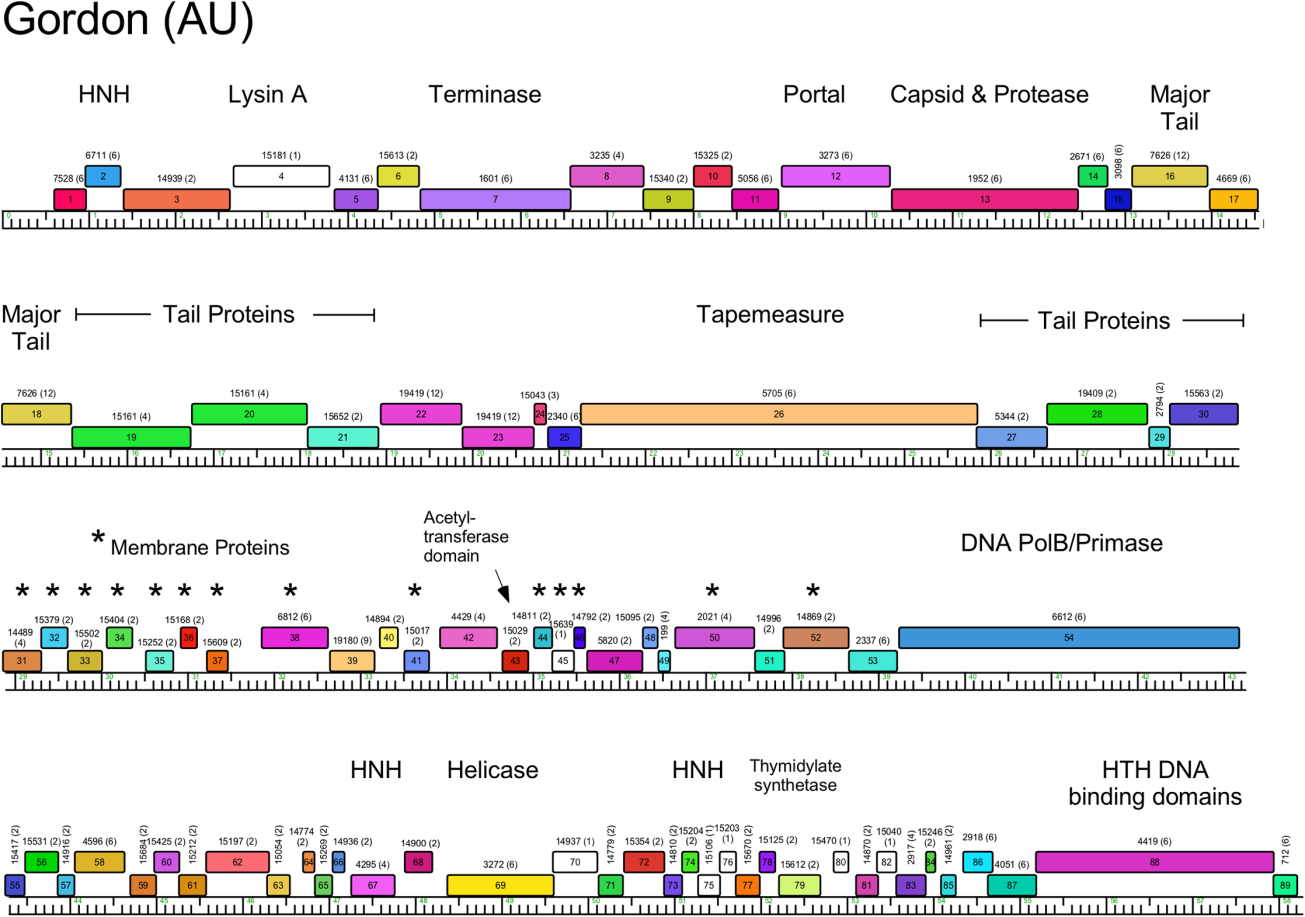
Genome organization of *Arthrobacter* phage Gordon, Cluster AU. See Fig 5 for details.

**Fig 9:**
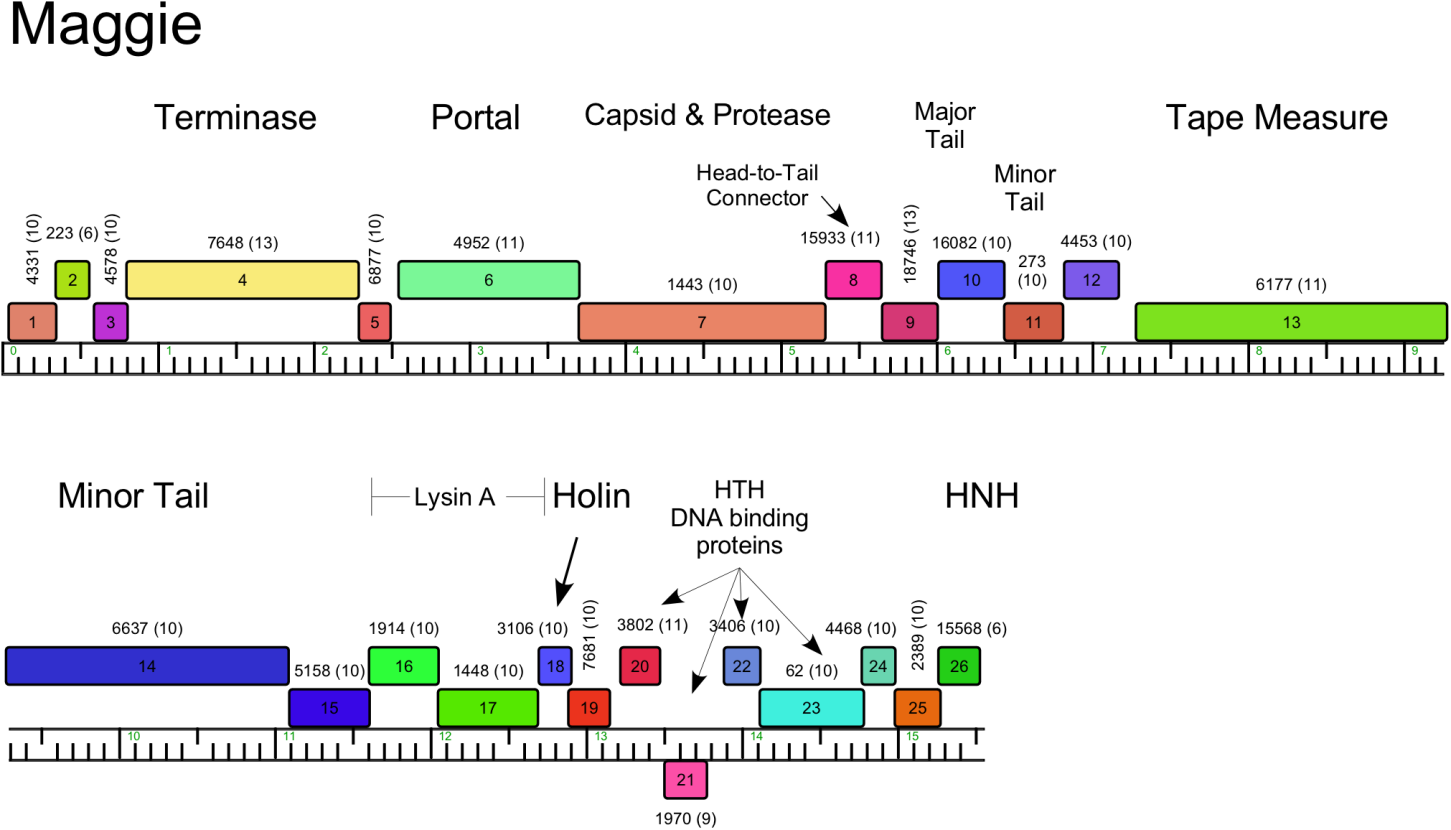
Genome organization of *Arthrobacter* phage Maggie, Cluster AN. See Fig 5 for details.

**Fig 10:**
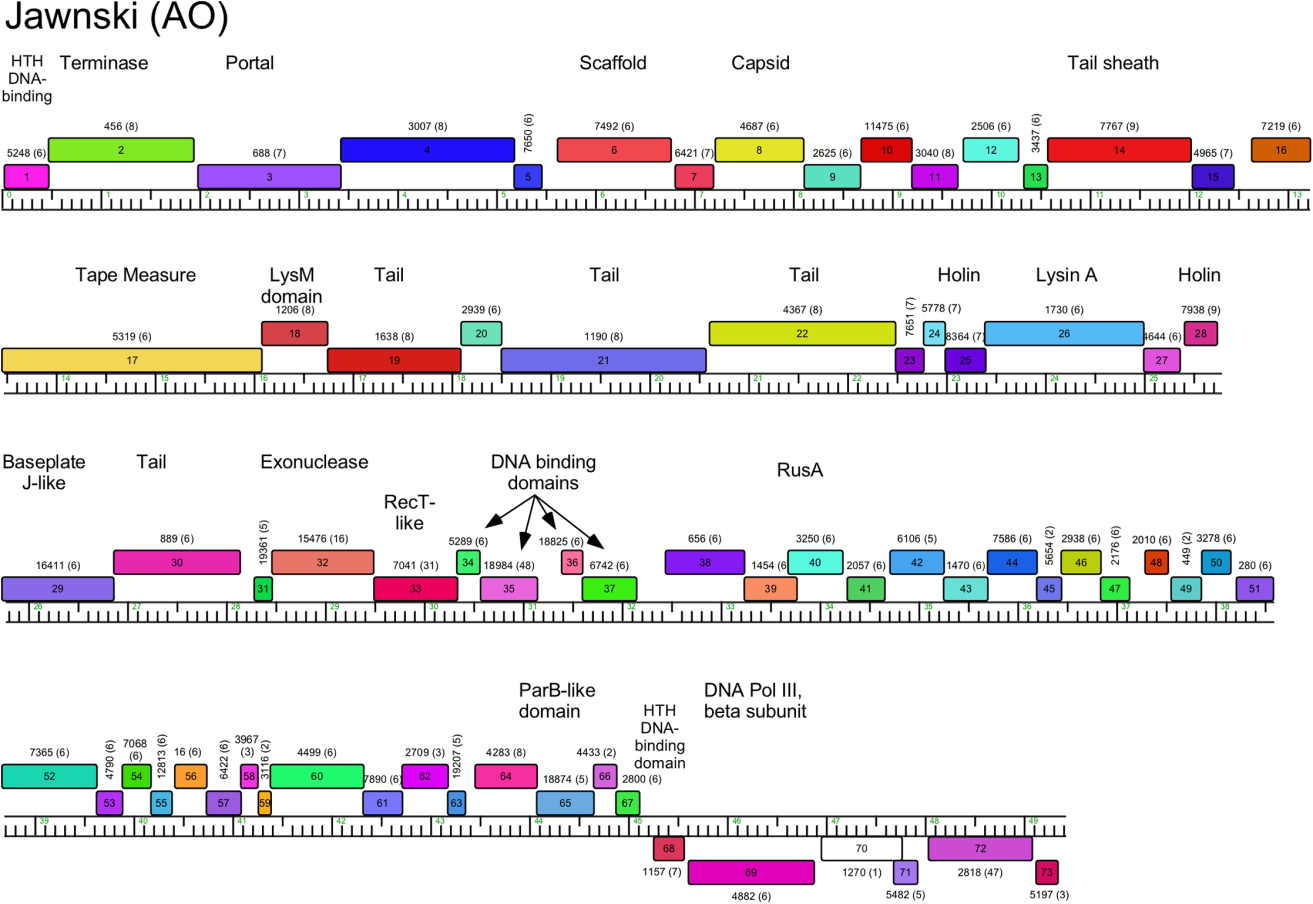
Genome organization of *Arthrobacter* phage Jawnski, Cluster AO. See Fig 5 for details.

**Fig 11:**
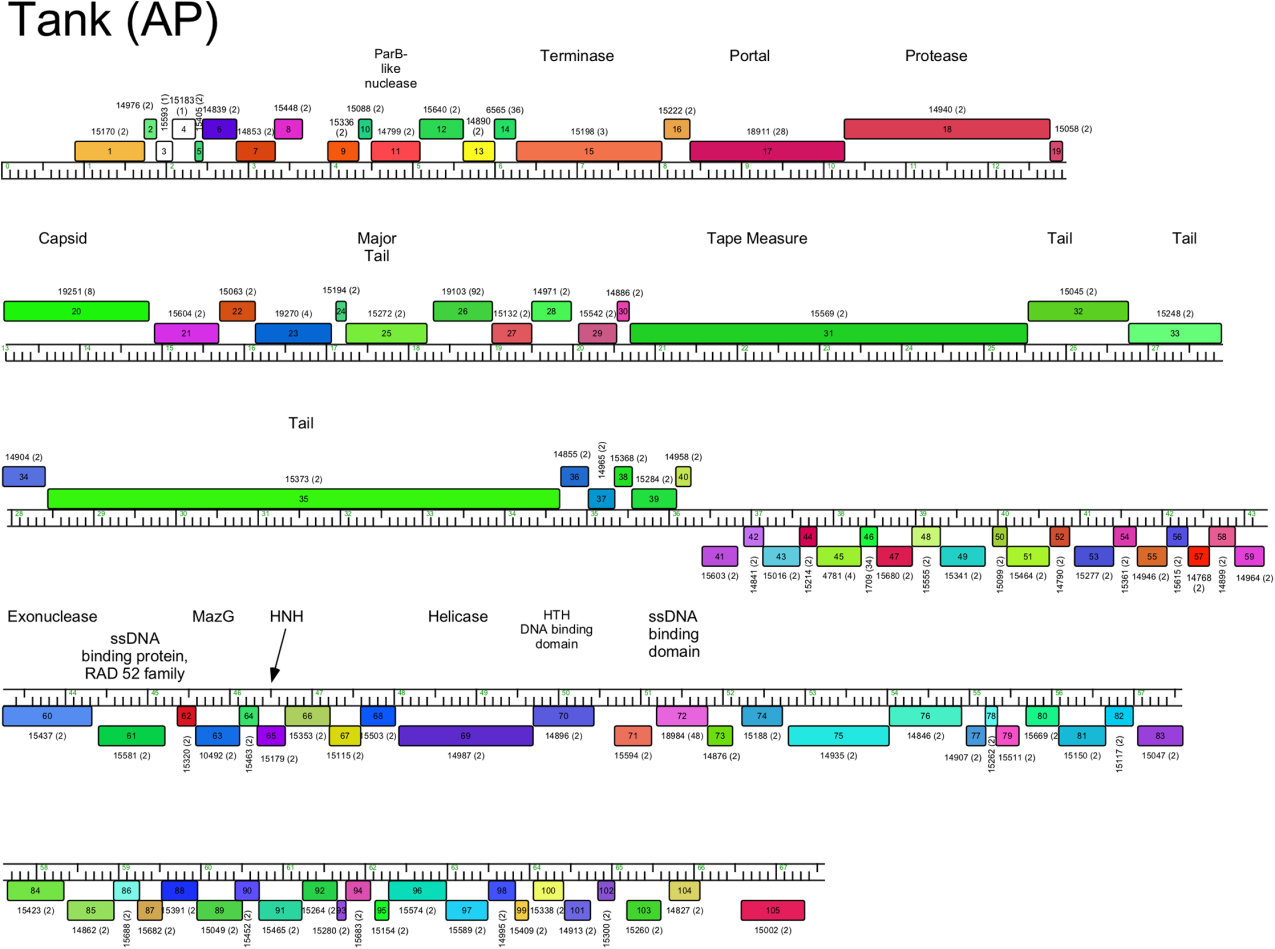
Genome organization of *Arthrobacter* phage Tank, Cluster AP. See Fig 5 for details.

**Fig 12:**
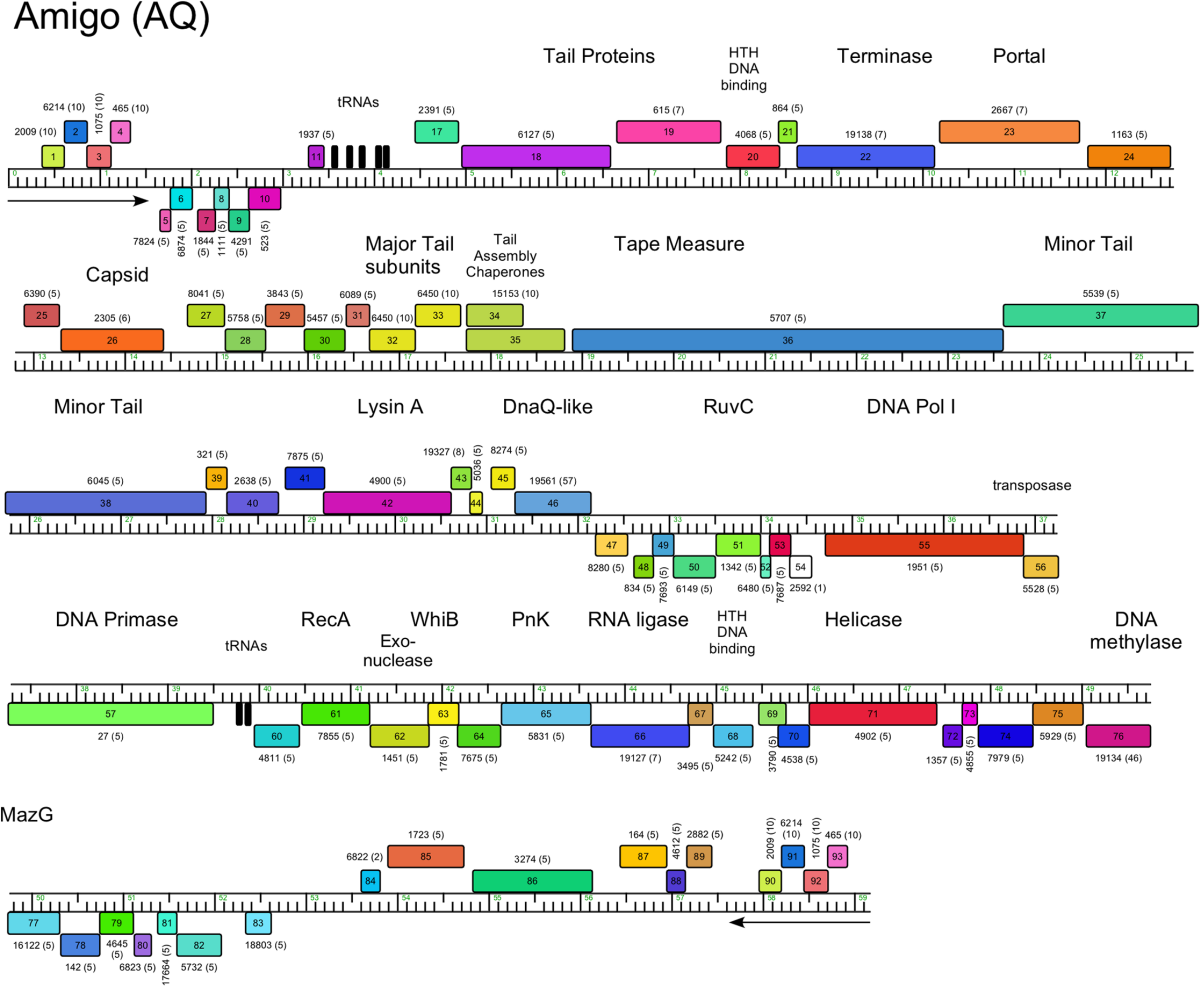
Genome organization of *Arthrobacter* phage Amigo, Cluster AQ. See Fig 5 for details.

**Fig 13:**
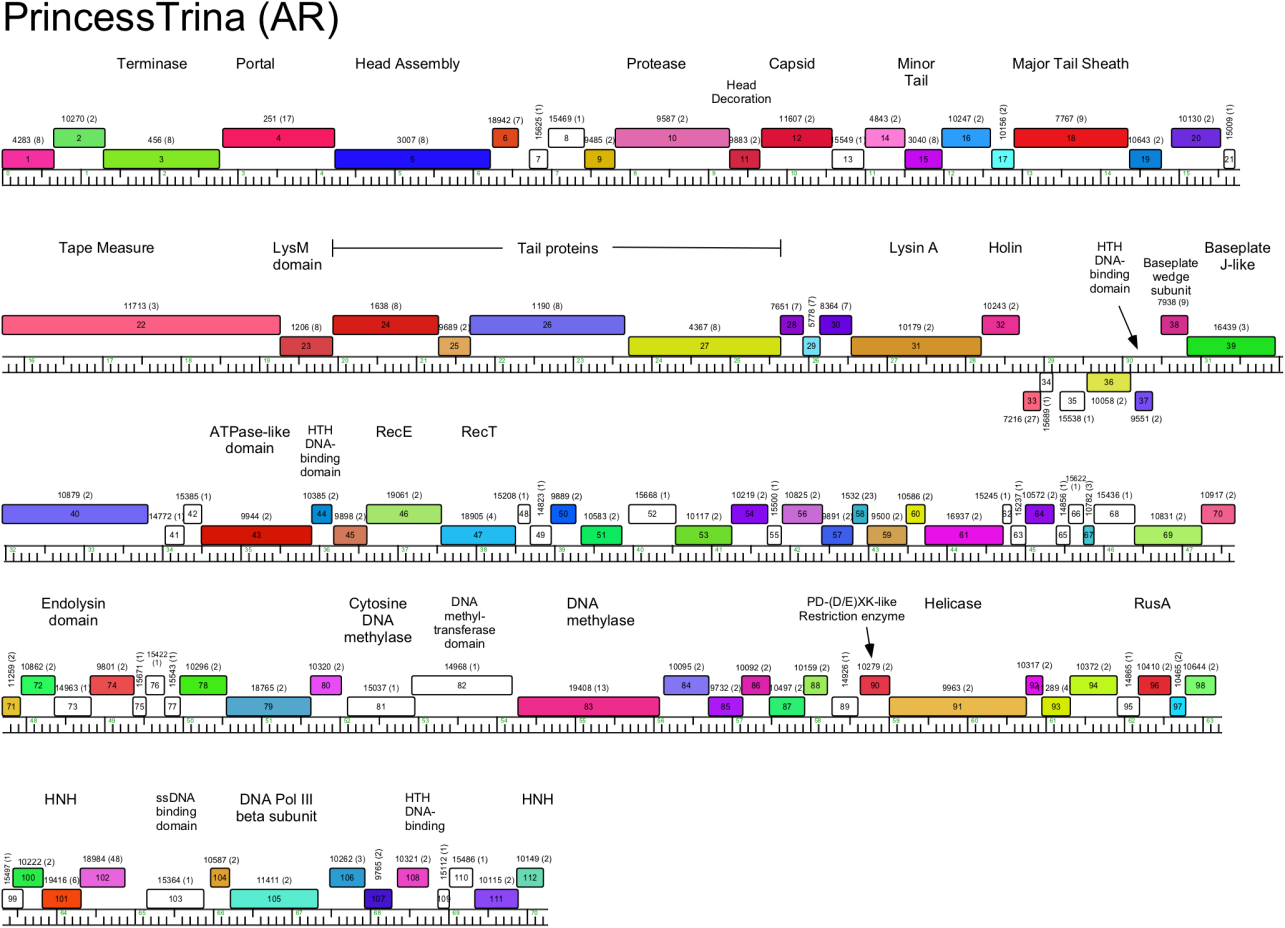
Genome organization of *Arthrobacter* phage PrincessTrina, Cluster AR. See Fig 5 for details.

**Fig 14:**
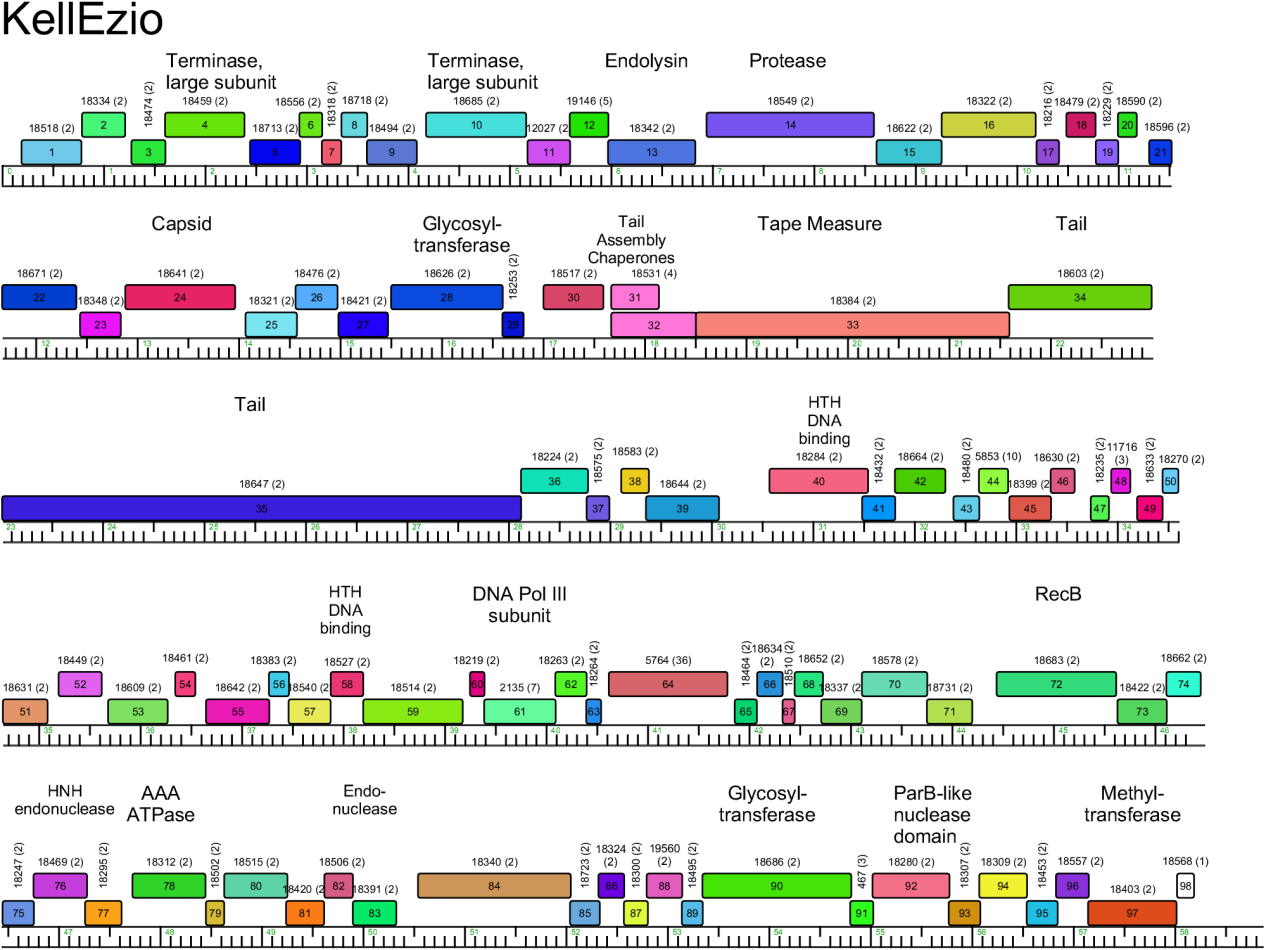
Genome organization of *Arthrobacter* phage KellEzio, Cluster AT. See Fig 5 for details.

**Fig 15:**
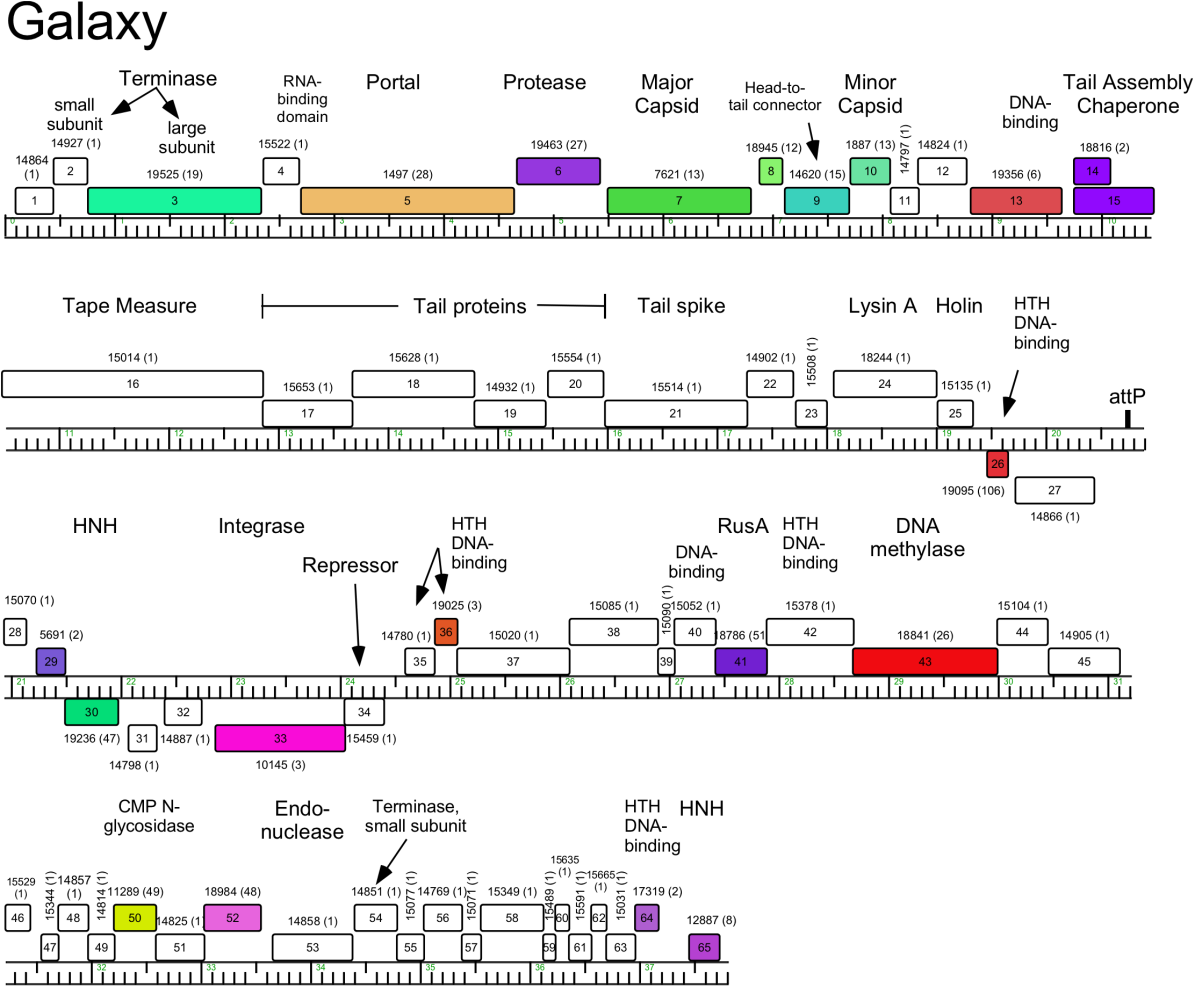
Genome organization of *Arthrobacter* phage Galaxy, Singleton. See Fig 5 for details.

The ten clusters and singletons Galaxy and Jasmine display a variety of genome organizations, reflecting variations on common architectural themes seen in other phages of the order *Caudovirales*. In general, the virion structure and assembly genes are organized with typical syntenic arrangement–terminase, portal, capsid maturation protease, scaffolding protein, major capsid protein, head-tail connectors, major tail subunit, tail chaperone proteins, tape measure protein, and minor tail proteins [32]–but are compactly organized in some genomes (e.g. Cluster AM) and are interrupted by non-structural genes in others (e.g. Cluster AL). In most of the genomes, the lytic functions are encoded immediately downstream of the virion genes, the exceptions being the Cluster AM and AU phages where the lytic gene is located upstream of the terminase, and in the Cluster AT phages, where it is between the terminase and capsid maturation protease genes; the remaining parts of the genomes include DNA metabolism genes and predicted regulatory functions. Galaxy is the only phage to encode an integrase, suggesting this it is temperate. Collectively, 62% of genes in these phages have unknown functions, and we note that the singletons Galaxy and Jasmine are replete with orphams, genes without homologues elsewhere in the Actinobacteriophages. We will briefly discuss the features of each cluster, and representative genomes maps are shown in Figs 5–15.

#### Cluster AK

The twelve Cluster AK phages (Table 1) are related to each other (Figs 2–4), with the virion genes in the left part of the genome and non-structural genes in the right part (Fig 5, S2 Fig). All genes are transcribed rightwards, with the exception of five leftwards-transcribed genes near the right end, one of which is a putative DNA binding protein (Fig 5). The portal and a Mu F-like protein are fused as a single gene (*6*) as shown in Fig 5.

#### Cluster AL

The two Cluster AL genomes are closely related and differ by 7–8 small insertions or replacements in the right portion of the genomes (Fig 6, S3 Fig). The genomes have been bioinformatically linearized 6.7 kbp upstream of the terminase large subunit gene, where there is a small non-coding gap. The genome organizations are unusual in that, although the virion structure and assembly genes have the canonical order, there are numerous and sometimes quite large insertions between them (Fig 6, S3 Fig). For example, in Laroye there are five genes inserted between the terminase large subunit (*10*) and portal genes (*16*), eight genes are inserted between the protease (*17*) and major capsid (*26*) genes, and 37 genes are found between the major capsid subunit (*26*) and major tail subunit gene (*64*) (where there are typically 5–6 head-tail connector genes). Although genes coding for ssDNA binding protein (*14*), adolase (*18*), RNase (*23*), and another DNA binding domain (*60*) are found in the insertions, most of the inserted genes are of unknown function. With these insertions, the virion structure genes span over 35 kbp, and more than 50% of the 60 kbp genome. The remaining parts of the genomes contain several genes whose functions can be predicted but are atypical in phage genomes, including an RNA helicase (*95*), an AIG2-like protein (gamma-glutamylcyclotransferase; *97*), an amidoligase (*98*), and a GTPase domain protein (*99*).

#### Clusters AM and AU

As noted above, the Cluster AM and AU genomes are distantly related, but share 25–30% of their genes, and the genome maps of Circum (AM) and Gordon (AU) are shown in Figs 7 and 8 and S4 Fig. The endolysin genes (Circum *7*, Gordon *4*) are located upstream of the terminase large subunit genes (Figs 7 and 8 and S3 Fig), as seen in Cluster A mycobacteriophages [37]. An unusual feature is the apparent fusion of the major capsid subunit and capsid maturation protease functions into a single gene (e.g. Circum *12*). This is reminiscent of previously described fusion proteins, such as the capsid and scaffold genes in *E*. *coli* HK97 [38] and the scaffold and protease fusions in phage Lambda [39].

Another unusual feature in the genomes of Cluster AM and AU phages is the presence of several small genes downstream of the tail genes, many of which encode putative membrane proteins. In Circum, 14 genes in the region of genes *25*–*61* encode proteins with between one and four membrane spanning domains, and 16 Gordon genes in the region of genes *31*–*52* (Figs 7 and 8) encode proteins with between one and five membrane spanning domains. The functions of these genes are unknown, but we note that similar arrays of putative membrane proteins are also present in *Rhodococcus* phages Pepy6 and Poco6 [6], and some of these share amino acid sequence similarity to Cluster AM and AU phages genes.

#### Cluster AN

The ten cluster AN phages are very closely related with small differences at their extreme left ends and some small regions of no sequence similarities (Fig 9 and S5 Fig). They have unusually small genomes for dsDNA phages, and are among the smallest of the *Siphoviridae* (Table 1). With an average of 15.5 kbp they are slightly larger than the smallest siphovirus genome reported, *Rhodococcus* phage RRH1 (14,270 bp) [40]. Much of the genome coding potential is occupied by the larger virion structure and assembly protein genes as shown in the map of Maggie (Fig 9), including a fused protease-capsid gene, similar to the gene fusions in Cluster AM and AU phages, but share little or no sequence similarity to Maggie gene *7*. Interestingly, the small *Rhodococcus* phage RHH1 has a similarly fused gene, and the predicted protein is a distant relative (27% amino acid identity) of Maggie gp7 (Fig 9). The non-structural genes (*20*, *21*, *22*, *23*), include those coding for four putative DNA binding proteins one of which (*21*) is the only leftwards transcribed gene. There are no genes coding for DNA metabolism functions, and these phages illustrate how few genes are required for propagation as a dsDNA tailed virus.

#### Cluster AO

The six Cluster AO phages share substantial genome similarity (Figs 2–4, S6 Fig) and a map of the Jawnski genome is shown in Fig 10. The virion structure and assembly genes are canonically ordered, but include a tail sheath and baseplate-like protein genes consistent with the contractile tail virion morphology (Fig 1); the lysis cassette appears to be inserted within the end of the tail gene operon (Fig 10). Jawnski codes for a RecET recombination system (genes *32* and *33*) and a beta subunit of DNA Pol III (*69*), but most of the non-structural genes are of unknown function.

#### Cluster AP

The two Cluster AP genomes, Tank and Wilde, are closely related with 5–6 small insertions and deletions relative to each other (Fig 11, S7 Fig). The genomes have direct terminal repeats and the virion structure and assembly genes are canonically ordered but include an unusually long minor tail gene (*35*, 6.5 kbp), which atypically exceeds the length of the tape measure protein gene (*31*, 4.8 kbp). The genome is organized into rightwards- and leftwards transcribed genes (*1*–*40* and *41*–*105*, respectively), which converge close to the center of the genome (Fig 11, S7 Fig). Most of the leftwards-transcribed genes are of unknown function, with the exceptions of those coding for a single-stranded DNA binding protein (*72*), a DNA helicase (*69*), a MazG-like protein (*63*), and a Rad52_Rad22 family recombinase (*61*) that likely functions together with a putative exonuclease (*60*).

#### Cluster AQ

The five Cluster AQ genomes also have long terminal direct repeats (1.5 Kbp), but which include four protein-coding genes of unknown function (Fig 12, S8 Fig). The organization of the virion structure and assembly genes is somewhat non-canonical with two large predicted tail genes upstream of the terminase large subunit gene as shown in the map of Amigo (Fig 12). A long operon of leftwards-transcribed gene (*47*–*83*) includes many with predicted DNA metabolism functions including DNA Pol I (*55*), RuvC (*51*), DNA Primase (*57*), RecA (*61*), DNA Helicase (*71*) and a DNA Methylase (*76*), as well as an RNA Ligase (*66*) and a polynucleotide kinase (*65*). The Cluster AQ phages are the only *Arthrobacter* phages encoding tRNA genes (Fig 12), each having seven tRNA genes with the exception of phage Rings, which has lost one of these.

#### Cluster AR

PrincessTrina and the previously described ArV1 [24] constitute Cluster AR and they share extensive nucleotide sequence similarity. Apart from five leftwards-transcribed genes (*33*–*37*), all of the genes are transcribed rightwards (Fig 13). The virion structure and assembly genes are canonically ordered but include major tail sheath (PrincessTrina *18*) and baseplate proteins (*39*) consistent with a contractile tail morphology; the lysis genes are located immediately downstream. Most of the non-structural genes are of unknown function, although several are predicted DNA metabolism genes including three putative DNA methylase genes (*81*, *82*, *83*); downstream, gene *90* codes for a PD-(D/E)XK-like restriction enzyme. Collectively, these genes may function as a restriction modification systems, or the DNA methylases could provide defense against host restriction systems.

#### Cluster AT

The two Cluster AT phages, KitKat and KellEzio, are closely related with 4–5 insertions and deletions relative to each other. All genes are transcribed in the rightwards direction (Fig 14, S9 Fig), and there are several unusual organizational features. First, there is an uncommonly long tail gene (*35;* 5.1 kbp) that exceeds the length of the tape measure gene (*33;* 3 kbp) reflecting a similar feature in the Cluster AP phages. Second, the endolysin gene (*13*) is located between the terminase large subunit and capsid maturation protease genes, a position unique to these Cluster AT phages. Third, there are two genes coding for products related to terminase large subunit genes (*4*, *10*). We also note the presence of two glycosyltranferase genes (*28*, *90*), one of which (*28*) is located between the capsid subunit and major tail subunit genes.

#### Singletons Galaxy and Jasmine

Galaxy’s genome is 37,809 bp with defined genome cohesive ends (Fig 15). Galaxy unusually has two genes (*2*, *54*) predicted to code for terminase small subunits. We note that several of the structural genes (e.g. *5*, *6*, *7*) have sequence similarity to some mycobacteriophages, a rare example of genes shared between mycobacteriophages and *Arthrobacter* phages. However, a high proportion of Galaxy genes are orphams (i.e. do not have relatives elsewhere in the Actinobacteriophage_692 database and shown as white boxes in Fig 15), a typical feature of singleton phages [10].

Galaxy is the only temperate phage among this group of *Arthrobacter* phages, and integrase (Int-Y) and repressor genes are predicted (*33* and *34*, respectively; Fig 15). Their organization is reminiscent of the mycobacteriophage integration-dependent immunity systems [41], but lack other common features such as recognizable degradation tags. Also, the *attP* site is not located within the repressor gene, and is positioned between genes *27* and *28* (coordinates 20,716–20,755) displaced by five genes from the integrase gene (*33*; Fig 15). The host genome contains two putative *attB* sites, located in identical tandemly repeated tRNA^met^ genes (*AUT_13455* and *AUT26_13460*). However, we have been unsuccessful in isolating stable Galaxy lysogens in *Arthrobacter* sp. ATCC21022, a similar scenario to that reported for Arthrobacter phage ArV2, which also has putative integrase and repressor genes, but for which stable lysogens could not be recovered [23].

**Fig 16:**
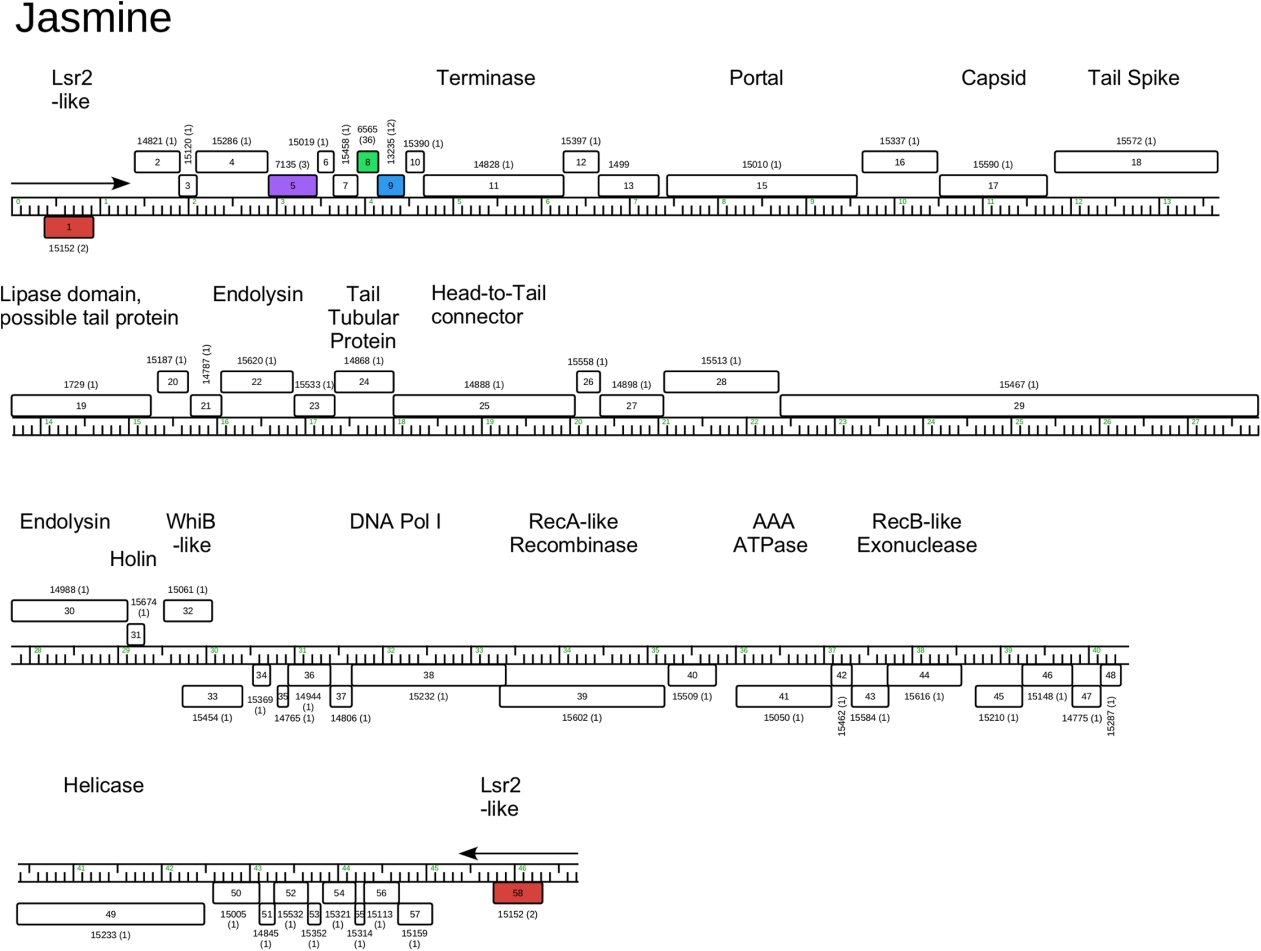
Genome organization of *Arthrobacter* phage Jasmine, Singleton. See Fig 5 for details.

The Jasmine genome is notable for the large number of orpham genes that lack relatives in the Actinobacteria database (Fig 16); only four of the 58 predicted genes have close relatives. It is the only sequenced Actinobacteriophage with a podoviral morphology (Fig 1), and the genome has 1,330 bp terminal direct repeats. Interestingly, the terminal repeat contains the complete coding region for an Lsr2-like gene, a distant relative to the Lsr2-like genes in several mycobacteriophages [42]. Database comparisons suggest the virion structure and assembly genes are coded in the left part of the genome (genes *11*–*29*), and include a putative tail spike gene (*18*; HHpred 99.81% probability score with the HK620 tail spike protein).

### Lysis functions

Phage lysis functions are of interest as they provide insights into the host cell wall that must be compromised for cell lysis. *Arthrobacter spp*. lack mycolic acids in their cell walls, and thus the complete absence of lysin B genes encoding esterases cleaving the linkage of mycolic acids to the cell wall [43] is not surprising. However, endolysin genes can be identified in most of the phages, and in most cases a closely linked putative membrane protein likely acts as a holin. Notable exceptions are the Cluster AP phages (Tank, Wilde) for which we have not been able to identify an endolysin gene. We note that there are several small genes at the left ends of the genomes coding for putative membrane proteins that are holin candidates (*3*, *5*, *6*), and it is plausible that one of the genes between the leftmost direct terminal repeat and the terminase gene codes for an endolysin that is not discovered by database searches. The *Arthrobacter* phage endolysins are highly diverse and modular (Table 2), reflecting the complex structures reported for the mycobacteriophage endolysins [44]. Some have three domains (peptidase, amidase, and cell wall binding domains; Clusters AL, AO, AR), whereas others have only subsets of these (Table 2). The phages in Cluster AN (e.g. Maggie) have the lysis functions coded in two separate genes (e.g. Maggie *16* and *17*); gp16 has the predicted peptidase activity and the amidase and cell wall binding activities are in gp17. We note that Jasmine has two genes (*22* and *30*) predicted to code for amidase functions, but *22* is located with the tail genes, and thus is more likely to be associated with phage infection than lysis.

**Table 2:** Endolysin domains.

				Endolysin domains		
Cluster	Phage	Gene	Length (aa)	HHPred match	phage match in HHPred	CDD match
AK	Korra	26	286	N-acetylmuramoyl-L-ala amidase	Prophage Lambdaba02, E4e-26	amidase-2, pfam01510
AL	Laroye	82	299	L-Ala-D-Glu peptidase	Enterobacter phage T5 lysozyme, E6e-12	no match
				peptidoglycan hydrolase	Pseudomonas phage PhiKZ lysin,E1.3e-05	no match
				peptidoglycan binding	Pseudomonas phage PhiKZ lysin, E4.9e-10	PG-binding-1, pfam01471
AM	Circum	5	309	N-acetylmuramoyl-L-ala amidase	Pseudomonas aeruginosa, E1.8e-23	M23 pfam01551
AN	Maggie	16	148	peptidase_M23	E1.2e-17	M23, pfam01551
		17	213	N-acetylmuramoyl-L-ala amidase	Staphylococcus phage GH15, E2.4e-28	PGRP, cI02712
AO	Jawnski	26	535	N-acetylmuramoyl-L-ala peptidase	Streptococcus phage C1, E6e-15	CHAP, pfam05257
				peptidoglycan hydrolase	Clostridium phage PHISM101, E7.7e-12	no match
				peptidoglycan binding, amidase	Prophage Lambdaba02, E2.94e-23	PGRP, cI02712
AP	Tank	none identified				
AQ	Amigo	42	464	Peptidoglycan peptidase	Streptococcus phage K, E1.5e-23	NLPC_60, cI21534
				N-acetylmuramoyl-L-ala amidase	Enterobacteria phage T7, E4e-24	PGRP, cd06583
AR	PrincessTrina	31	551	peptidase	Staphylococcus phage K, E8.4e-24	NLPC_60, cI21534
				muramidase, peptidoglycan hydrolase	Clostridium phage PHISM101, E9.8e-38	GH25 muramidase, pfam01183
				peptidoglycan binding	Thermus thermophilus, E1.4e-19	LysM (3 domains), cd00118
AT	KellEzio	13	286	peptidase	Staphylococcus phage K, E8.4e-24	NLPC_60, cI21534
				N-acetylmuramoyl-L-ala amidase	Paenibacillus polymyxa hydrolase, E0.026	no match
AU	Gordon	4	369	amidase-2 domain, hydrolase	Staphylococcus phage GH15 lysin, E1.6e-23	PGRP, cd06583
				peptidoglycan binding	Bacteriophage CP-7 lysozyme, E3.8e-14	CPL-7 lysozyme, cI07020 (4 domains)
Singleton	Galaxy	24	312	peptidoglycan binding, amidase	Enterobacteria phage T7 lysozyme, E2e-22	PGRP, cI02712
				peptidoglycan binding	Thermus thermophilus, E7.6e-14	LysM (2 domains), cd00118
Singleton	Jasmine	22	270	peptidoglycan binding, amidase	Staphylococcus phage GH15 lysin, E2.9e-21	PGRP, cI02712
		30	436	Lysozyme-like muramidase	Staphylococcus aureus lysozyme, E8.8e-31	NLPC_60, cI21534

### Evolutionary relationships

**Fig 17:**
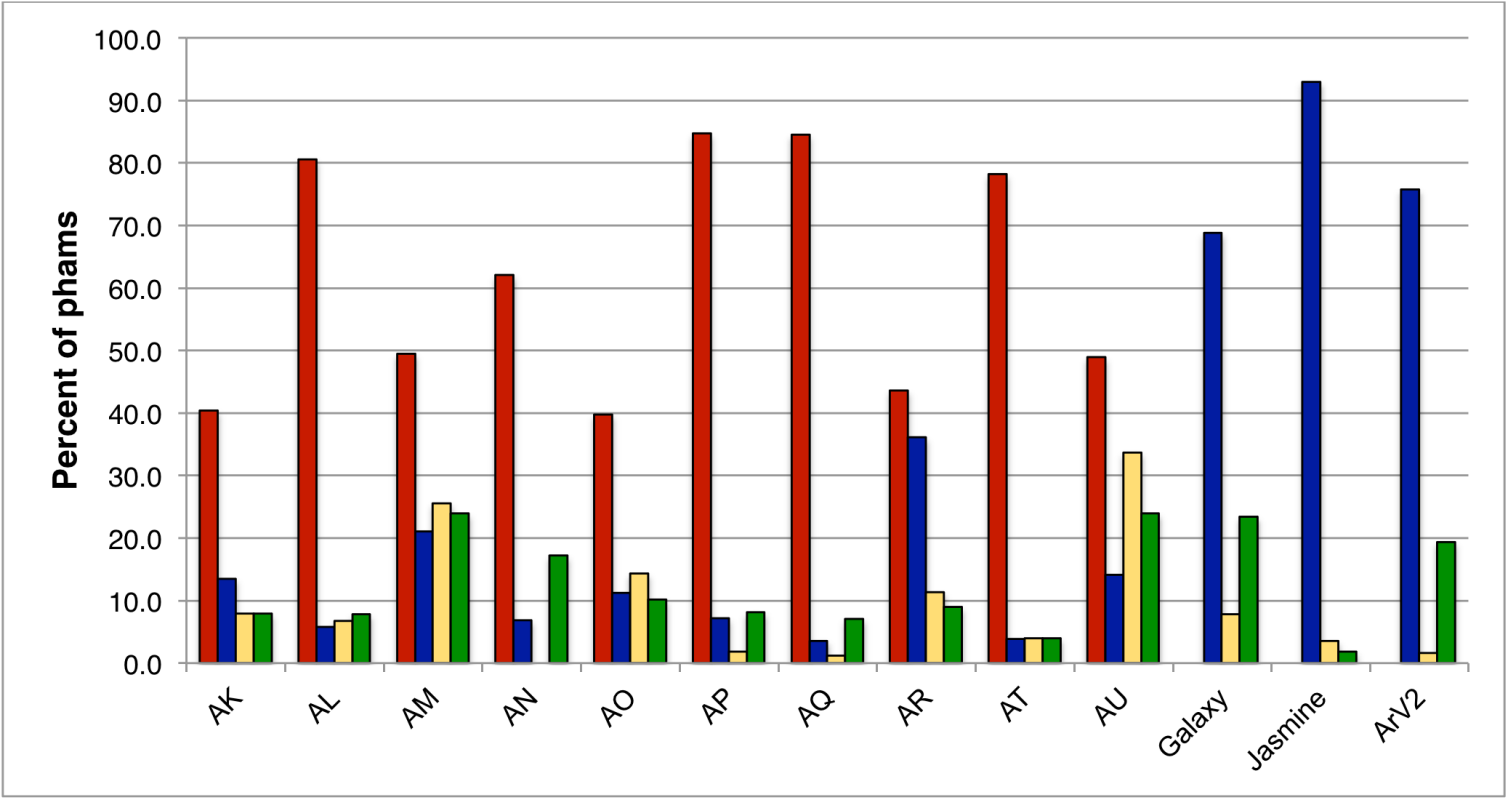
Cluster diversity and inter-cluster relationships. Intra-cluster diversity was determined by the percent of cluster-identifier phams (phams present in all members of a cluster and not found in phages of other clusters, red bars, not calculated for singleton phages), and the percent of orphams (phams present in only one phage, with no homologues in the database, blue bars). Inter-cluster relationships are shown as the proportion of phams present in each *Arthrobacter* phage cluster that are also present in at least one phage of another *Arthrobacter* cluster (yellow bars) or in at least one phage infecting a host other than *Arthrobacter* (green bars). The number of phages in each cluster is indicated in parentheses below the cluster name.

This collection of sequenced Arthrobacter phages provides insights into their spectrum of diversity relative to phages of other hosts, and how they are related specifically to phages of other Actinobacterial hosts. We note that the *Arthrobacter* phages distribute into a similar number of clusters and singletons (10, 2, respectively) identified when only 60 mycobacteriophage genomes had been sequenced, which formed 9 clusters and 5 singletons [32]. This reflects a greater overall diversity than seen with phages of *Propionibacterium acnes* [45]. To investigate this further we examined the distributions of gene phamilies (phams) representing groups of related proteins (see Material and Methods). The 3272 genes coding in the 48 genomes are grouped into a total of 1067 phams (S4 Table), 273 of which (26%) are orphams with no close relatives in the database; these are especially prevalent in the singletons Galaxy and Jasmine (Figs 15–17). The proportions of “cluster-associated” phams–those present in all cluster members but not present in any other cluster–varies substantially among the clusters (Fig 17) indicating the degrees to which the overall diversity varies among the clusters; it does not correlate with the numbers of cluster members (Fig 17).

We also examined the extent to which the *Arthrobacter* phages are exchanging genes between clusters, or are relatively isolated. This is reflected in the numbers of phams in each cluster that are also present in at least one phage in another *Arthrobacter* cluster (Fig 17, S5 Table). For six clusters (AK, AL, AN, AP, AQ and AT) fewer than 10% of gene phamilies are in this category, reflecting relatively high levels of cluster isolation. Clusters AM and AU have more of these shared phams in part because they share about 25% of their genes with each other. Cluster AO and AR likewise share about 20% of their genes, and these relationships are also reflected in the shared branches in the Splitstree phylogeny shown in Fig 3. We note that similar cluster isolation measures for the mycobacteriophages range from 16–77% with an average of 60.8% [10].

Interestingly, the number of phams present in phages of Actinobacterial hosts other than *Arthrobacter* (103 of 1052 phams, 9.7%; S6 Table) is similar to the numbers shared between *Arthrobacter* phage clusters (Fig 17). Thus, the clusters are not only genetically well isolated from each other, but the genes that are shared are just as likely to be shared by non-*Arthrobacter* phages as they are by other *Arthrobacter* phages. We note, however, that there is considerable variation among the clusters in the patterns of shared genes. For example, the Cluster AU phages share more genes with other *Arthrobacter* phages than non-*Arthrobacter* phages, whereas in Cluster AN, AP, AQ and the singleton Galaxy, the opposite pattern is observed (Fig 17). Moreover, the genes are not shared with the phages of any one different host, but are broadly distributed, including phages of other Corynebacteriales hosts (*Mycobacterium*, *Gordonia*, *Rhodococcus*, *Corynebacterium*, *Tsukamurella*) as well as *Streptomyces*, *Propionibacterium*, and other Micrococcales hosts *Clavibacter* and *Microbacterium* (S6 Table). Over half of the shared genes (53/101) are in Actinobacteriophages other than those infecting *Mycobacterium*, even though those are only 10% of the non-*Arthrobacter* Actinobacteriophages. The most striking relationship is that between Clusters AM and AU with *Rhodococcus* phages Poco6 and Pepy6 (S10 Fig), with more than 20 shared genes distributed across the entire genome spans, most with more than 50% amino acid identity (S6 Table); there is also weak but evident nucleotide sequence similarity (S10 Fig**)**.

**Fig 18:**
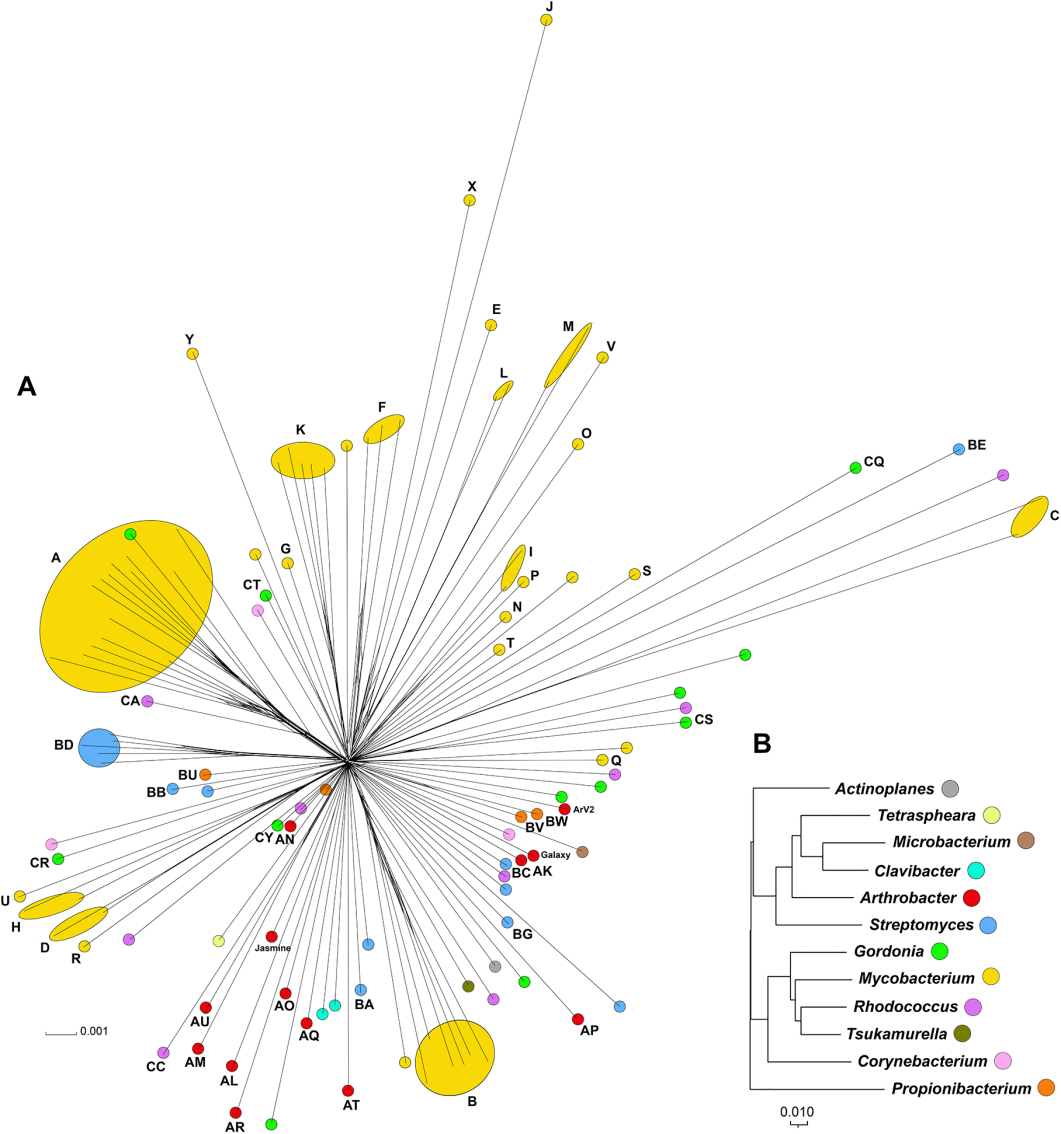
Comparison of phage shared gene content and host phylogeny. **A.** One representative phage genome from each cluster including singletons were assigned a value reflecting the presence or absence of each pham in the database, and the genomes were compared and displayed using Splitstree [36]. Clusters are labeled with the cluster name, and singleton phages isolated in Arthrobacter are identified; all others are singleton phages isolated in other hosts. Colors correspond to bacterial host genera in panel B. The scale bar indicates 0.001 substitutions/site. **B.** Phylogenetic tree derived from 16S rRNA sequences from representative bacteria from each phage host genus in the database. Evolutionary analyses were conducted in MEGA7 [46] using the Neighbor-Joining method with gaps eliminated. The scale bar indicates 0.01 base substitutions per site. The 16S rRNA sequences (GenBank accession numbers in parentheses) were from *Actinoplanes* sp. SE50/110 (CP003170), *Arthrobacter* sp. ATCC 21022 (CP014196), *Clavibacter michiganensis* (AB299158), *Corynebacterium vitaeruminis* DSM 20294 (NR_121721), *Gordonia terrae* 3612 (CP016594), *Microbacterium foliorum* strain 122 (CP019892), *M*. *smegmatis* mc^2^ 155 (Y08453), *Propionibacterium acnes* ATCC 11828 (CP003084), *Rhodococcus erythropolis* PR4 (AP008957), *Streptomyces griseus* strain DSM 40236 (AP009493), *Tetraspheara remsis* strain 3-M5-R-4 (DQ447774), *Tsukamurella paurometabola* DSM 20162 (NR_074458). This tree mirrors the phylogeny of 90 actinobacteria based on 16S rRNA gene sequences as described previously [47] but also includes *Actinoplanes* and *Tetraspheara*.

Interestingly, these relationships do not obviously mirror the phylogeny of the actinobacterial hosts. *Arthrobacter* is more closely related to *Streptomyces* that it is to *Mycobacteria*, *Gordonia*, or *Rhodococcus* (Fig 18), but only nine *Arthrobacter* phage phams are shared with *Streptomyces* phages (of which there are 32 in the database used). In contrast, 36 *Arthrobacter* phage phams are shared with *Rhodococcus* phages (of which there are 16 in the database used). Although the numbers of phages available for these types of analyses are still small, there is little evidence of a correlation between shared gene content of representative phages from each actinobacteriophage cluster and phylogenetic proximity of their hosts (Fig 18, S7 Table). We also tested 21 Arthrobacter phages for their abilities to infect 29 different Actinobacterial hosts, including nine other *Arthrobacter* species (see Materials and Methods). None of the *Arthrobacter* phages tested infected any of these strains, and no mutants with expanded host range were identified. These narrow host preferences reflect those reported previously for ArV2 [24] and ArV1 [23].

### Concluding remarks

Here we have described 46 newly isolated phages of *Arthrobacter* sp. ATCC21022 and compared their genomic sequences. They are richly diverse in morphotype and genotype, with 12 distinct lineages forming ten clusters and two singletons. These clearly represent an under-sampling of the broader population-at-large of phages infecting this strain, and the diversity of the large collection of mycobacteriophages suggests that the sequenced *Arthrobacter* phage collection will need to be expanded 10-20-fold to reflect better their genomic diversity. Given the narrow host range of these phages, we also predict that phages isolated on other *Arthrobacter* strains will reveal phage genomic lineages not previously described. The dearth of temperate phages among those described here is somewhat surprising, as they represent the majority of phages isolated on *M*. *smegmatis* [10] and on *Gordonia terrae* (unpublished observations). Because all of these phages were isolated from similar environments, the relative preponderance of temperate and lytic phages appears to be a function of the host used for isolation, rather than different environmental parameters, although we note that metagenomic studies suggest that temperate phages are more prevalent in environments with higher bacterial densities [48]. The roles of the hosts in directing evolution of phage lifestyles remains obscure, but isolation and genomic characterization of large sets of phages on hosts within the Actinobacteria will hopefully illuminate this question.

## Materials and methods

### Bacterial strains and media

All phages were isolated on *Arthrobacter* species, ATCC strain 21022. Either LB media (L-agar base) or PYCa media (containing per 1 liter volume: 1.0 g Yeast extract, 15 g Peptone, 2.5 mL 40% Dextrose, and 4.5 ml 1M CaCl_2_) were used for phage isolation and amplification.

### *Arthrobacter* phage isolation, propagation, and virion analysis

All phages were obtained from soil samples with permissions granted (S1 Table). For the soil enrichment protocol, 1–2 grams of soil were incubated at 30°C with *Arthrobacter sp*. in PYCa or LB medium supplemented with 1–4.5 mM CaCl_2_ a and *Arthrobacter sp*. host for 2–5 days. These enriched soil samples were filtered with 0.22 μm—0.45 μm filters and the filtrates were introduced to a pure culture of *Arthrobacter sp*. Some soil samples were not enriched with host bacteria prior to performing a plaque assay. For these samples, the soil samples were treated with phage buffer (10mM Tris-HCL, pH 7.5; 10mM MgSO_4_; 68.5mM NaCl; 1mM CaCl_2_), shaken vigorously, filtered, and plated directly on solid overlays containing 0.35% agar and *Arthrobacter* host and incubated at 30°C for 16–48 hours. For both the enriched soil samples and the direct soil samples, individual plaques were purified. Once plaque purified, high-titer *Arthrobacter* phage stocks and plate lysates were obtained using methods described previously for Mycobacterial hosts [26]. Phage particles were spotted onto formvar and carbon-coated 400 mesh copper grids, rinsed with distilled water and stained with 1% uranyl acetate. Images were taken using a FEI Morgagni transmission electron microscope. Measurements were performed on at least 3 particles for each phage.

### Genome sequencing, annotation, and analysis

*Arthrobacter* phages were isolated, sequenced, and annotated in the PHIRE or SEA-PHAGES programs. Phage genomes were shotgun-sequenced using either 454, Ion Torrent, or Illumina platforms to at least 20-fold coverage. Shotgun reads were assembled *de novo* with Newbler versions 2.1 to 2.9. Assemblies were checked for low coverage or discrepant areas, and targeted Sanger reads were used to resolve weak areas and identify genome ends. Genomes were annotated using DNA Master (http://cobamide2.bio.pitt.edu), GLIMMER [49], GeneMark [50], BLAST, HHPred [51], and Phamerator [29]. Actinobacteriophage_692 is the Phamerator database used for the analyses of this project. Further analyses included Dot plot (Gepard) [35], Splitstree [36], kAlign [52], and TMHMM transmembrane helix prediction (http://www.cbs.dtu.dk/services/TMHMM/). All genome sequences are publicly available at phagesdb.org and in GenBank.

### Host range testing

High titer lysates of 21 *Arthrobacter* phages (Bennie, Joann, Korra, Pumancara, Wayne, Laroye, Salgado, Circum, Maggie, Moloch, Toulouse, Jawnski, Martha, Sonny, TaeYoung, Wilde, Amigo, KellEzio, Kitkat, Gordon,and Galaxy) were serially diluted in phage buffer and 10 μl of ten-fold dilutions were spotted onto 29 Actinobacteria hosts lawns prepared from the following strains: *Arthrobacter atrocyaneus B-2883*, *Arthrobacter citreus B-1258*, *Arthrobacter globiformis B-2979*, *Arthrobacter globiformis B-2880*, *Arthrobacter humicola B-24479*, *Arthrobacter pascens B-2884*, *Arthrobacter viscosus B-1973*, *Arthrobacter viscosus B-1797*, *Arthrobacter sulfureus B-14730*, *Tsukamurella wrastlaviensis* NRRL B-16958, *Tsukamurella sunchanesis* NRRL 24668, *Tsukamurella pauramutabola* NRRL 16960, *Rhodococcus erythroplois* NRRL 1574, *M*. *smegmatis* mc^2^155, *Mycetocola saprophilus* NRRL B-24119, *Microbacterium hominus* NRRL B-24220, *Microbacterium foliorum* NRRL B-24224, *Microbacterium aerolatum* NRRL B-24228, *Kocuria* species (Hatfull lab collection), *Kocuria* 68 (Dutton lab collection), *Gordonia westfalica* NRRL 16540, *Gordonia terrae* NRRL 3612, *Gordonia rubripertincta* NRRL 24152, *Corynebacterium vitaeruminis* ATCC 10234, *Corynebacterium glutamicum* ATCC 14020, *Corynebacterium flavescens* ATCC 10340, *Brevibacterium* samyangense NRRL B-41420, *Brevibacterium fuscum* NRRL B-14687, and *Brachybacterium* sp. 113 (Dutton lab collection). The plates were incubated at room temperature with the exception of *M*. *smegmatis* mc^2^155, which was incubated at 37°C, and *Gordonia terrae* and *Microbacterium foliorum*, which were incubated at 30°C. Plates were examined after 24 and 48 hours of incubation.

## Supporting information

S1 Fig Location of phages on United States map. (PDF)

S2 Fig Pairwise alignment of 11 Cluster AK *Arthrobacter* phages. (PDF)

S3 Fig Pairwise alignment of 2 Cluster AL *Arthrobacter* phages. (PDF)

S4 Fig Pairwise alignment of 2 Cluster AM and 2 Cluster AU *Arthrobacter* phages. (PDF)

S5 Fig Pairwise alignment of 10 Cluster AN *Arthrobacter* phages. (PDF)

S6 Fig Pairwise alignment of 6 Cluster AO *Arthrobacter* phages. (PDF)

S7 Fig Pairwise alignment of 2 Cluster AP *Arthrobacter* phages. (PDF)

S8 Fig Pairwise alignment of 5 Cluster AQ *Arthrobacter* phages. (PDF)

S9 Fig Pairwise alignment of 2 Cluster AT *Arthrobacter* phages. (PDF)

S10 Fig Relationship between Clusters AM, AU, CC. (PDF)

S1 Table Location of soil source and permissions. (PDF)

S2 Table Measurements of head diameter and tail length in electron micrographs. (PDF)

S3 Table Average nucleotide identity (ANIs) of 48 *Arthrobacter* phages. (XLSX)

S4 Table Pham data summary. (XLSX)

S5 Table Phams shared between *Arthrobacter* phage clusters. (PDF)

S6 Table Phams shared between *Arthrobacter* phages and phages isolated on other Actinobacteria hosts. (XLSX)

S7 Table Gene content analysis data for Fig 18A. (NEX)
