# Supplementary material for: Tales of diversity: Genomic and morphological characteristics of forty-six Arthrobacter phages
Source: PLoS One. 2017 Jul 17;12(7):e0180517. doi: 10.1371/journal.pone.0180517 (PMC5513430; doi:10.1371/journal.pone.0180517)
Supplement: S1 Fig — (PDF) [file pone.0180517.s001.pdf]

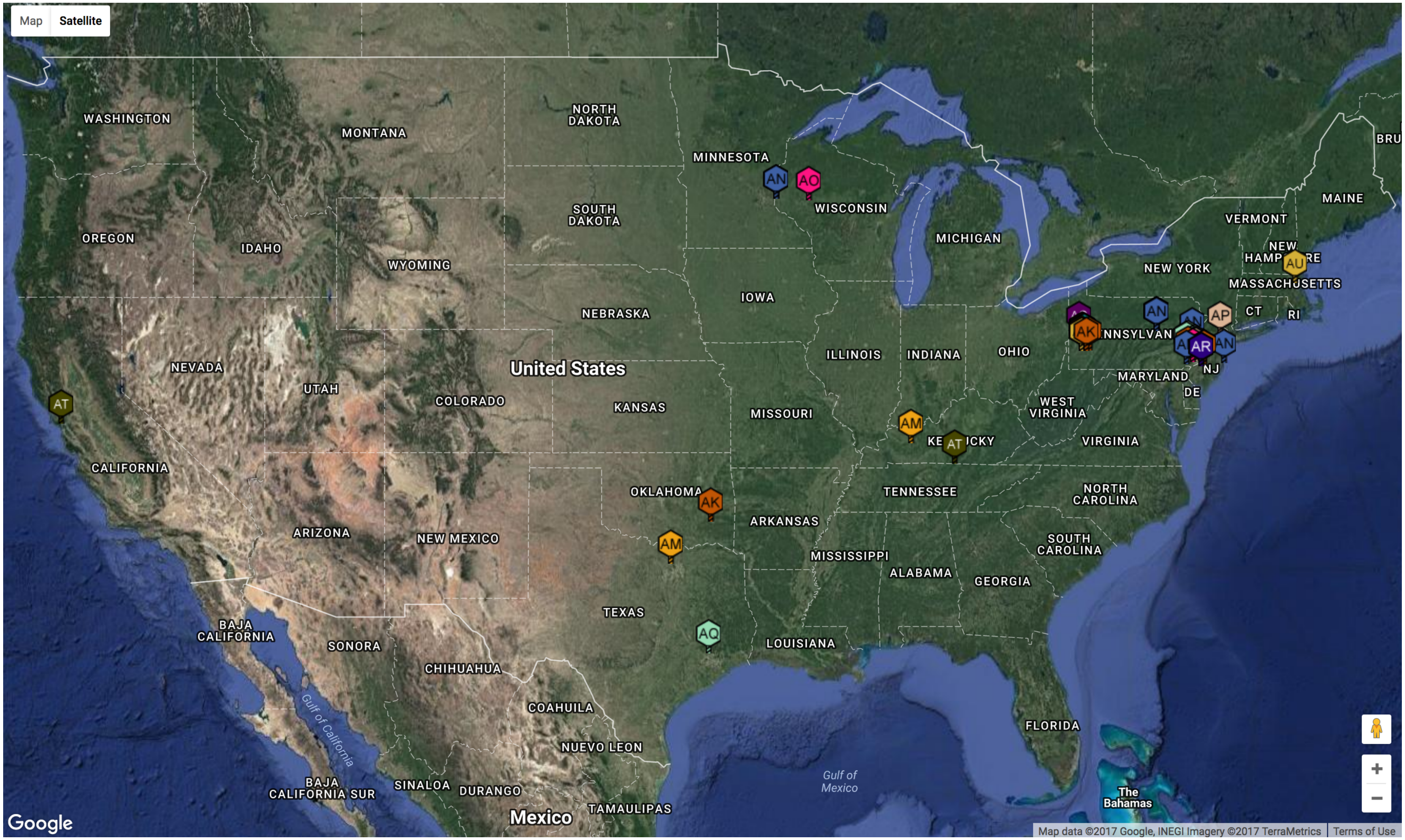

S1 Figure. Location of phages on United States map. Each pin represents the location of the soil sampled for phage isolation.

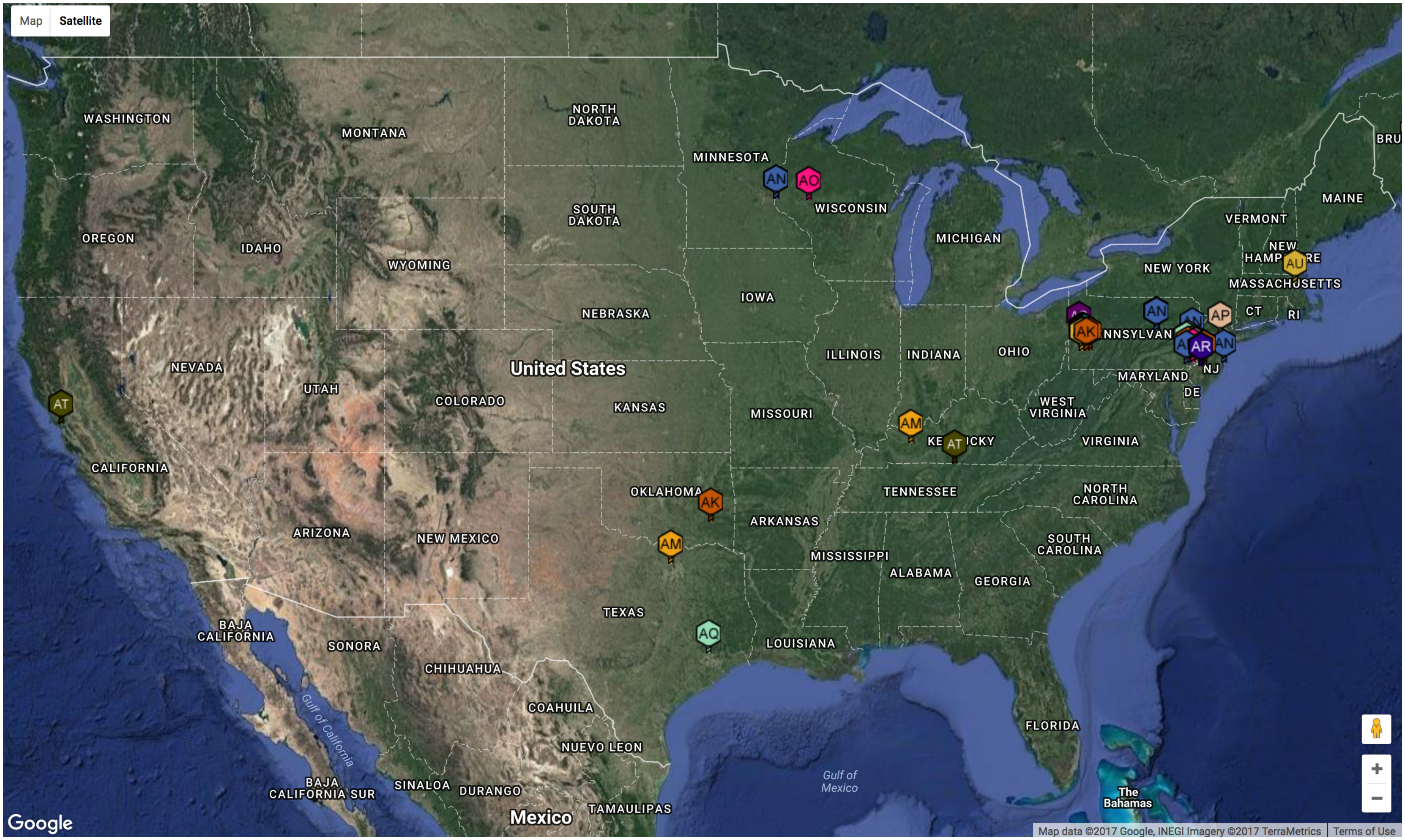

S1 Figure. Location of phages on United States map. Each pin represents the location of the soil sampled for phage isolation.
