# Supplementary figures and images for: Tales of diversity: Genomic and morphological characteristics of forty-six Arthrobacter phages
Source: PLoS One. 2017 Jul 17;12(7):e0180517. doi: 10.1371/journal.pone.0180517 (PMC5513430; doi:10.1371/journal.pone.0180517)

Bennie

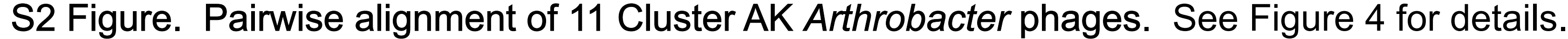

Supplement: S2 Fig — (PDF) [file pone.0180517.s002.pdf]
