# Supplementary material for: Tales of diversity: Genomic and morphological characteristics of forty-six Arthrobacter phages
Source: PLoS One. 2017 Jul 17;12(7):e0180517. doi: 10.1371/journal.pone.0180517 (PMC5513430; doi:10.1371/journal.pone.0180517)
Supplement: S3 Fig — (PDF) [file pone.0180517.s003.pdf]

Cluster AL

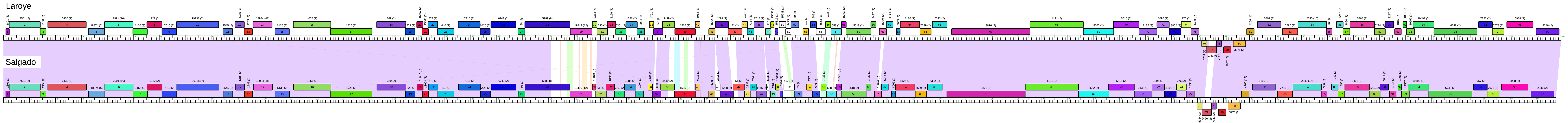

S3 Figure. Pairwise alignment of 2 Cluster AL *Arthrobacter* phages. See Figure 4 for details.
