# Supplementary material for: Tales of diversity: Genomic and morphological characteristics of forty-six Arthrobacter phages
Source: PLoS One. 2017 Jul 17;12(7):e0180517. doi: 10.1371/journal.pone.0180517 (PMC5513430; doi:10.1371/journal.pone.0180517)

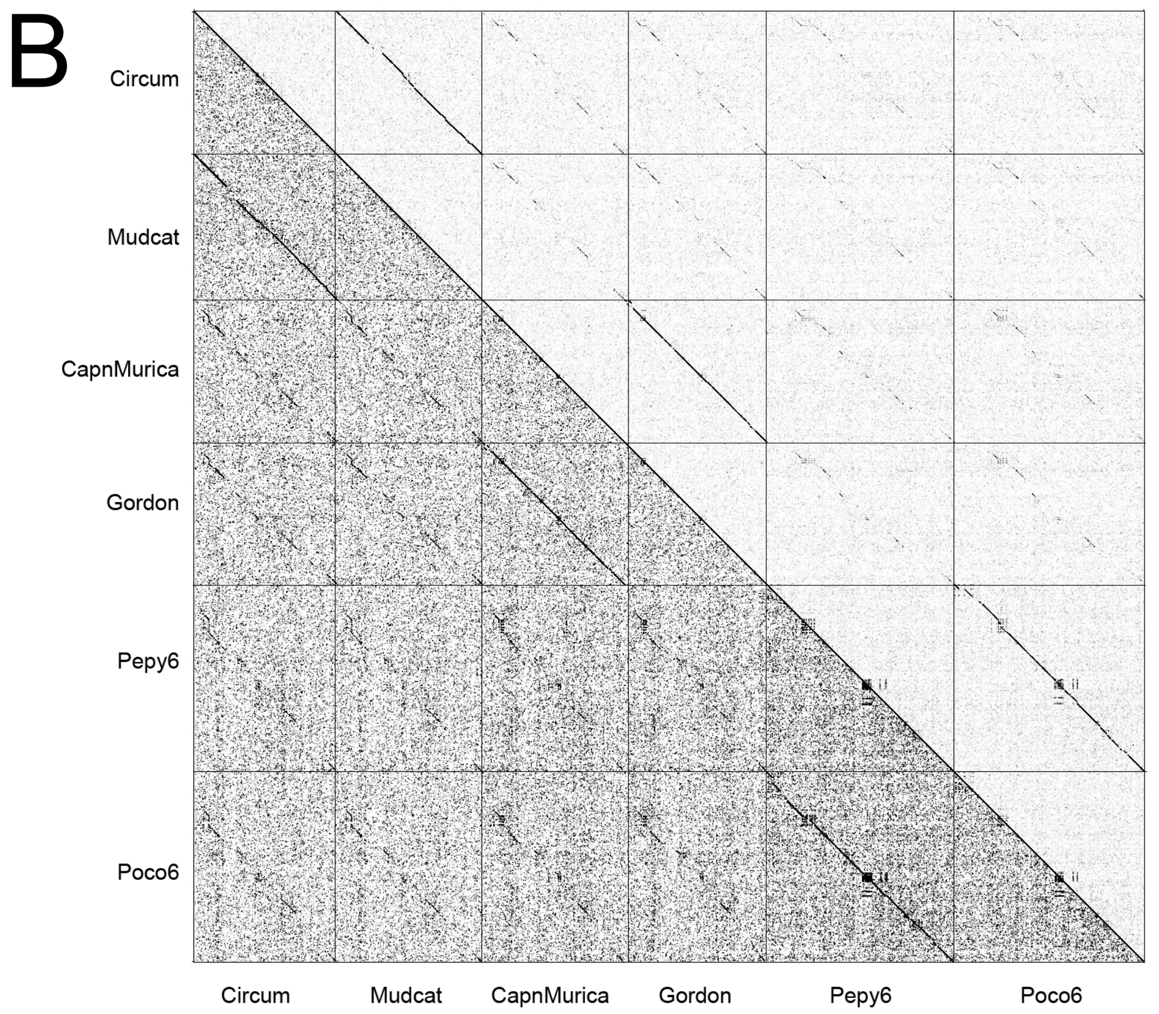

S10 Figure. Relationship between Clusters AM, AU, and CC. **A.** Pairwise alignment of *Arthrobacter* phages Circum (Cluster AM), Gordon (Cluster AU), and *Rhodococcus* phages Pepy6 and Poco6 (Cluster CC), displayed using Phamerator. See Figure 4 fore details. Asterisks designate transmembrane proteins. **B.** Dotplot showing nucleotide comparisons of phages Circum and Mudcat (Cluster AM), Gordon and CapnMurica (Cluster AU), and Pepy6 and Poco6 (Cluster CC). See Figure 4 for details.

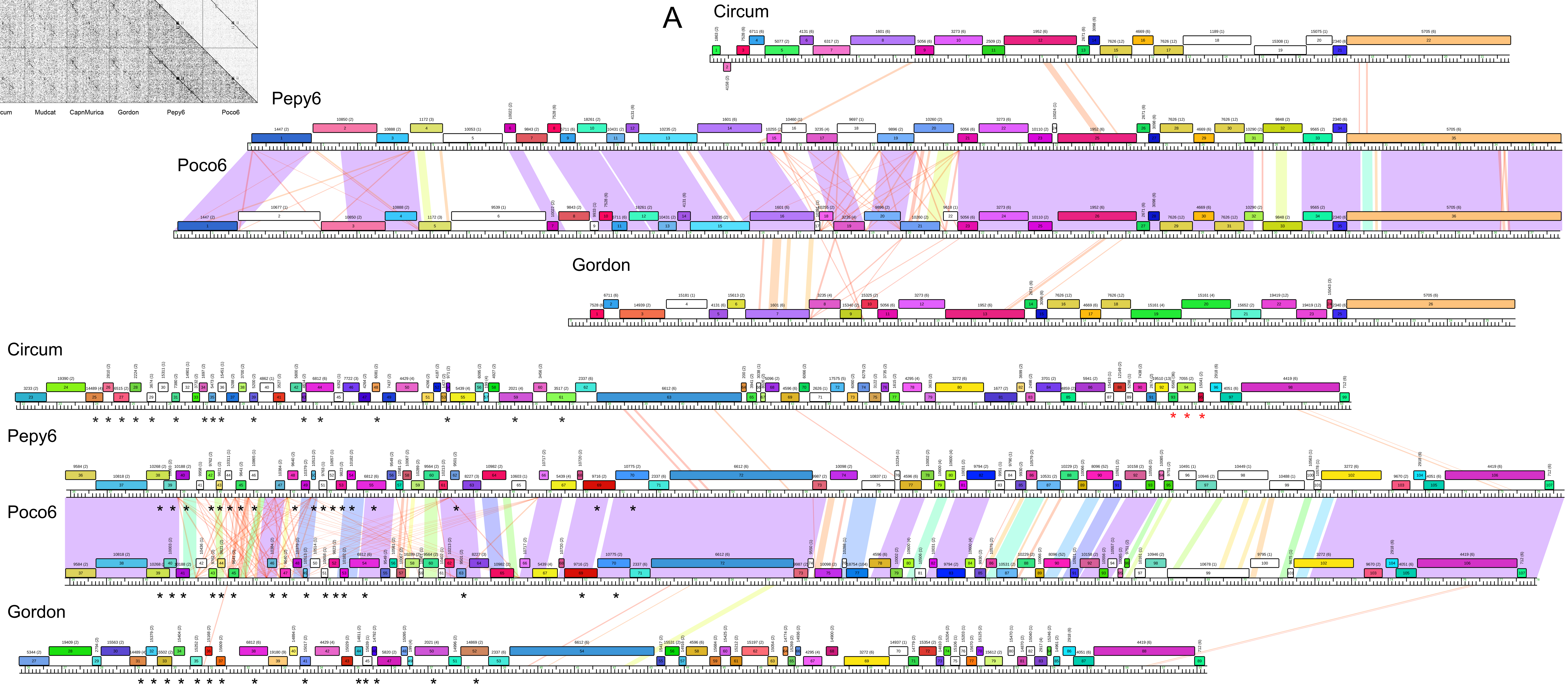

Supplement: S10 Fig — (PDF) [file pone.0180517.s010.pdf]
