# Supplementary material for: Tales of diversity: Genomic and morphological characteristics of forty-six Arthrobacter phages
Source: PLoS One. 2017 Jul 17;12(7):e0180517. doi: 10.1371/journal.pone.0180517 (PMC5513430; doi:10.1371/journal.pone.0180517)
Supplement: S1 Table — (PDF) [file pone.0180517.s011.pdf]

Table S1: Sample Locations & Permissions

Phage	Location	Permit/permissions
Amigo	Private home, Spring, Tx	permission granted
Anansi	Private home, Phoenixville, PA	permission granted
BarretLemon	private home, Chippewa Falls, WI	permission granted
Bennie	South Park, PA	no permit required
Brent	private home, Broomall, PA	Permission granted
CapnMurica	Carnegie Mellon University	no permission needed
Circum	U of North Texas	no permission needed
Decurro	public property, Lewisburg, PA	no permit required
DrRobert	U of Pittsburgh	no permission needed
Galaxy	Seneca Valley High School	no permission needed
Glenn	U of Pittsburgh	no permission needed
Gordon	South Park, PA	no permit required
Gorgeous	Private home, Lafayette Hill, PA	permission granted
HunterDalle	public property, Laurel Springs, NJ	no permit required
Immaculata	Immaculata University	no permission needed
Jasmine	Schenley Park, Pittsburgh PA	no permit required
Jawnski	Schenley Park, Pittsburgh PA	no permit required
Jessica	public property, Lewisburg, PA	no permit required
Joann	private property, Clayton, OK	permission granted
KellEzio	public property, Burkesville, KY	no permit required
Kitkat	private home, Greenbrae, CA	permission granted
Korra	Overlook Park, Bethel Park, PA	no permit required
Laroye	U of Pittsburgh	no permission needed
Maggie	Lehigh University	no permission needed
Martha	private property, Pittsburgh, PA	permission granted
Moloch	public space, Pittsburgh, PA	no permit required
Mudcat	public property, Central City, KY	no permit required
Muttlie	private home, West Chester, PA	permission granted
Preamble	private property, Radnor, PA	permission granted
PrincessTrina	private home, Laurel Springs, NJ	permission granted
Pumancara	private property, Pittsburgh, PA	permission granted
RAP15	private property, Pittsburgh, PA	permission granted
Rings	public park, Radnor, PA	no permit required
Salgado	U of Pittsburgh	no permission needed
Sandman	public beach, Seaside Heights, NJ	no permit required
Sonny	U of Pittsburgh	no permission needed
SorJuana	public property, Royersford, PA	no permit required
Stratus	public space, Radnor, PA	no permit required
TaeYoung	U of Pittsburgh	no permission needed
Tank	Saint Joseph's Univ	no permission needed
Toulouse	private home, Hudson, WI	permission granted
TymAbreu	private home, Hudson, WI	permission granted
Vulture	public property, Marlton, NJ	no permit required
Wayne	private home, N. Huntingdon, PA	permission granted
Wilde	private home, Montclair, NJ	permission granted
Yank	public property, Lewisburg, PA	no permit required
