# Supplementary material for: Tales of diversity: Genomic and morphological characteristics of forty-six Arthrobacter phages
Source: PLoS One. 2017 Jul 17;12(7):e0180517. doi: 10.1371/journal.pone.0180517 (PMC5513430; doi:10.1371/journal.pone.0180517)
Supplement: S2 Table — (PDF) [file pone.0180517.s012.pdf]

Supplementary Table S2: Measurements of virion head diameters and tail lengths

Phage	Length	Cluster	Head (nm)	Tail (nm)
Bennie	43074	AK	56.8±1.4	130.3±9.3
Laroye	60005	AL	56.4±1.5	133.6±6.1
Circum	58353	AM	73.7±1.3 x 50.5±2.2	228.1±4.0
Maggie	15556	AN	37.6±2.3	96.4±5.8
Jawnski	49149	AO1	54.3±6.4	117.4±6.2
Martha	51027	AO2	58.4±1.9	118.0±9.2
Wilde	68203	AP	62.2±1.5	236.1±1.0
Amigo	59173	AQ	61.4±1.7	242.3±13.3
KitKat	58560	AT	61.4±2.4	149.6±11.5
Gordon	58279	AU	58.8±5.3	237.6±12.9
PrincessTrina	70265	AR	57.5±1.4	176.8±17.5
Galaxy	37809	Singleton	55.1±3.4	120.2±7.9
Jasmine	46723	Singleton	59.8±2.9	10.3±0.9
