# Supplementary material for: Tales of diversity: Genomic and morphological characteristics of forty-six Arthrobacter phages
Source: PLoS One. 2017 Jul 17;12(7):e0180517. doi: 10.1371/journal.pone.0180517 (PMC5513430; doi:10.1371/journal.pone.0180517)
Supplement: S5 Table — (PDF) [file pone.0180517.s015.pdf]

Table S5. Phams shared between Arthrobacter phage clusters

						Number of phages in each cluster containing pham												
						AK	AL	AM	AN	AO	AP	AQ	AR	AT	AU	Galaxy	Jasmine	ArV2
					# of cluster members:	12	2	2	10	6	2	5	2	2	2	1	1	1
pham	# members	# Arthro members	shared clusters	representative gene	function													
199	4	4	AM, AU	Circum_gp57	NKF[1]			2							2			
456	8	8	AO, AR	Jawnski_gp2	terminase, large subunit					6			2					
467	3	3	AO, AT	KitKat_gp94	NKF					1				2				
712	6	4	AM, AU	Circum_gp99	NKF			2							2			
1190	8	8	AO, AR	Jawnski_gp21	minor tail					6			2					
1206	8	8	AO, AR	Jawnski_gp18	LysM domain					6			2					
1601	6	4	AM, AU	Circum_gp8	terminase, large subunit			2							2			
1887	13	13	AK, Galaxy	Korra_gp12	NKF	12										1		
1952	6	4	AM, AU	Circum_gp12	capsid & capsid maturation protease			2							2			
2021	4	4	AM, AU	Circum_gp59	NKF			2							2			
2233	7	7	AK, AL	Korra_gp3	NKF	6	1											
2337	6	4	AM, AU	Circum_gp62	NKF			2							2			
2340	6	4	AM, AU	Circum_gp21	NKF			2							2			
2671	6	4	AM, AU	Circum_gp13	NKF			2							2			
2917	4	4	AO, AU	Sonny_gp67	NKF					2					2			
2918	6	4	AM, AU	Circum_gp96	hydrolase			2							2			
3007	8	8	AO, AR	Jawnski_gp4	NKF					6			2					
3040	8	8	AO, AR	Jawnski_gp11	NKF					6			2					
3098	6	4	AM, AU	Circum_gp14	NKF			2							2			
3272	6	4	AM, AU	Circum_gp80	ATP-dependent helicase			2							2			
3273	6	4	AM, AU	Circum_gp10	portal			2							2			
4051	6	4	AM, AU	Circum_gp97	NKF			2							2			
4131	6	4	AM, AU	Circum_gp6	NKF			2							2			
4206	12	12	AL., AL	Korra_gp55	HTH DNA binding domain	10	2											
4283	8	8	AO, AR	Jawnski_gp64	NKF					6			2					
4295	4	4	AM, AU	Circum_gp78	HNH endonuclease			2							2			
4367	8	8	AO, AR	Jawnski_gp22	minor tail					6			2					
4419	6	4	AM, AU	Circum_gp98	HTH DNA binding domain			2							2			
4429	4	4	AM, AU	Circum_gp50	NKF			2							2			
4596	6	4	AM, AU	Circum_gp69	NKF			2							2			
4669	6	4	AM, AU	Circum_gp16	NKF			2							2			
5056	6	4	AM, AU	Circum_gp9	NKF			2							2			
5705	6	4	AM, AU	Circum_gp22	tape measure			2							2			
6565	36	5	AM, AP, Jasmine	Circum_gp93	NKF			2			2						1	
6612	6	4	AM, AU	Circum_gp6	NKF			2							2			
6711	6	4	AM, AU	Circum_gp4	HNH endonuclease domain			2							2			
6812	6	4	AM, AU	Circum_gp44	NKF			2							2			
7135	3	3	AL, Jasmine	Laroye_gp71	NKF		2										1	
7528	6	4	AM, AU	Circum_gp3	NKF			2							2			
7626	12	8	AM, AU	Circum_gp15, Circum_gp17	major tail			4							4			
7651	7	7	AO, AR	Jawnski_gp23	NKF					6			1					
7767	9	8	AO, AR	Jawnshi_gp14	tail sheath					6			2					
7938	9	8	AO, AR	Jawnski_gp28	NKF					6			2					
8364	7	7	AO, AR	Jawnski_gp25	holin					6			1					
10782	3	3	AR, AT	PrincessTrina_gp67	NKF								2	1				
11716	3	3	AR, AT	KitKat_gp50	NKF								1	2				
12887	8	2	Galaxy, ArV2	Galaxy_gp55	NKf											1		1
14489	4	4	AM, AU	Circum_gp25	NKF			2							2			
15043	3	3	AL, AU	Salgado_gp30	NKF		1								2			
18984	48	13	AL, AO, AP, AR, Galaxy	Laroye_gp14	ssDNA binding domain		2			6	2		2			1		
19138	7	7	AL, AQ	Laroye_gp10	terminase, large subunit		2					5						
19180	9	3	AK, AU	Joann_gp51	NKF	1									2			
19419	12	12	AK, AL, AU	Korra_gp2	NKF	6	2								4			
19463	27	13	AK, Galaxy	Korra_gp7	capsid maturation protease	12										1		
19525	19	13	AK, Galaxy	Korra_gp5	terminase, large subunit	12										1		
19560	2	2	AR, AT	KellEzio_gp88	hydrolase domain								1	1				
[1]NKF, No known function																		
